# Vaccine protection by *Cryptococcus neoformans* Δ*sgl1* is mediated by γδ T cells via TLR2 signaling

**DOI:** 10.1038/s41385-022-00570-3

**Published:** 2022-10-13

**Authors:** Tyler G. Normile, Timothy H. Chu, Brian S. Sheridan, Maurizio Del Poeta

**Affiliations:** 1 Department of Microbiology and Immunology, Stony Brook University, Stony Brook, NY 11794 USA; 2 Division of Infectious Diseases, School of Medicine, Stony Brook University, Stony Brook, NY 11794 USA; 3 Veterans Administration Medical Center, Northport, NY 11768 USA

## Abstract

We previously reported that administration of *Cryptococcus neoformans* Δ*sgl1* mutant vaccine, accumulating sterylglucosides (SGs) and having normal capsule (GXM), protects mice from a subsequent infection even during CD4^+^ T cells deficiency, a condition commonly associated with cryptococcosis. Here, we studied the immune mechanism that confers host protection during CD4^+^T deficiency. Mice receiving Δ*sgl1* vaccine produce IFNγ and IL-17A during CD4^+^ T (or CD8^+^ T) deficiency, and protection was lost when either cytokine was neutralized. IFNγ and/or IL-17A are produced by γδ T cells, and mice lacking these cells are no longer protected. Interestingly, ex vivo γδ T cells are highly stimulated in producing IFNγ and/or IL-17A by Δ*sgl1* vaccine, but this production was significantly decreased when cells were incubated with *C. neoformans* Δ*cap59/*Δ*sgl1* mutant, accumulating SGs but lacking GXM. GXM modulates toll-like receptors (TLRs), including TLR2. Importantly, neither *Δsgl1* nor Δ*cap59/*Δ*sgl1* stimulate IFNγ or IL-17A production by ex vivo γδ T cells from TLR2^−/−^ mice. Finally, TLR2^−/−^ animals do not produce IL-17A in response to Δ*sgl1* vaccine and were no longer protected from WT challenge. Our results suggest that SGs may act as adjuvants for GXM to stimulate γδ T cells in producing IFNγ and IL-17A via TLR2, a mechanism that is still preserved upon CD4^+^ T deficiency.

## Introduction

Invasive fungal infections (IFI) result in ~1.5 million deaths each year and have become a global health concern^1,2^. These infections are primarily caused by environmental fungi, which mainly infect immunocompromised individuals, such as HIV/AIDS patients^3–5^, solid organ transplant recipients^6–8^, or those taking medication to control chronic diseases^9–12^. *Cryptococcus neoformans* is one of these environmental fungi with a key fungal virulence characteristic: the ability to grow and replicate at human body temperature of 37 °C. The emergence of cryptococcal infections in more temperate climates by tropically-restricted *C. neoformans* species suggests a role for climate change, particularly global warming, in increasing environmental fungal infections^13–16^. Together with the continual rise in the global immunocompromised population, the frequency of cryptococcosis and other IFI is expected to significantly increase in the future.

Upon inhalation of environmental cryptococcal spores, immunocompetent hosts normally control the infection, which often remains asymptomatic, likely forming lung granulomas where fungal cells remain dormant^17–19^. Immunocompromised individuals that fail to contain the initial infection or manage the lung granuloma will experience uncontrolled fungal replication in the lungs, which may lead to life-threatening meningoencephalitis if the fungus disseminates to the central nervous system^20–22^. Recent epidemiological studies reported an incidence of ~220,000 new cryptococcal cases/year with a mortality rate of ~180,000 deaths/year^5,23^.

The high rate of mortality upon extrapulmonary cryptococcosis in immunocompromised individuals can be attributed in part to the poor efficacy, host toxicity, and induced fungal resistance to current antifungal therapeutics as well as to the total lack of anticryptococcal vaccines^24–27^. Although there has been ample research into the development of a fungal vaccine (reviewed in refs. ^26,28,29^), none have advanced past the pre-clinical research stage. A major hurdle to overcome in the development of a vaccine against *C. neoformans* is the fact this pathogen infects mostly immunocompromised individuals with low or no CD4^+^ T cells^3,11,30^. In addition, vaccine studies should be carried out in animal models that resemble the immunodeficiency associated with cryptococcosis (e.g., lacking CD4^+^ T cells)^23,29,31^.

We have previously reported that the deletion of the sterylglucosidase 1 gene (*SGL1*) in *C. neoformans* produced an avirulent mutant strain (Δ*sgl1*) that accumulates large quantities of sterylglucosides (SGs) whose levels are undetectable in the *C. neoformans* wild-type (WT) strain^32^. SGs are glycolipids found in fungi, plants, and algae, but rarely in bacteria and animals^33–35^. Prior to our studies, data available on their biological functions were limited to plants or the fungus *Pichia pastoris*. Our physio-pathological studies provided the first evidence on the key role of Sgl1 on fungal virulence^32,36^, and our recent structural studies will enable the rational design of new antifungal agents targeting Sgl1^37^.

The immunological function of SGs was first suggested in 1996 by Bouic and colleagues^33^. They reported that the immune response, in particular the response of murine T helper cells in vitro, was affected by the administration of a certain plant SG, sitosterolin (a mixture of sitosterol/sitosterol β-glucoside). More importantly, the secretion of Th1 cytokines, such as IL-2 and IFNγ, was increased^33^. Later, Lee et al. found that *Candida albicans*-infected mice treated with plant-derived β-sitosterol glucosides (daucosterol) survived longer than untreated mice and had greater numbers of activated splenic lymphocytes compared to untreated mice^38^. β-sitosterol glucosides may also play a protective role in the septic process by mitigating inflammation in a rat model of sepsis^39^. Lastly, the effect of β-sitosterol glucosides on the immune response was also observed in a human study^40^. The daily administration of plant β-sitosterol glucosides (when combined with regular anti-tuberculosis treatment) increased Th1 lymphocyte proliferation and promoted the recovery of patients with pulmonary tuberculosis. Overall, these studies strongly suggest that SGs are potent immunomodulators but the mechanism by which they stimulate the host immune response remains largely unknown.

When we observed that *C. neoformans* Δ*sgl1* was avirulent and all mice were alive and cleared the mutant, we re-infected them with WT *C. neoformans*. We observed complete (100%) host protection, suggesting that *C. neoformans* Δ*sgl1* mutant can be used as a live, attenuated vaccine^32^. Subsequent studies showed that *C. neoformans* Δ*sgl1* provided complete host protection even during CD4^+^ T cell deficiency^41,42^. We also identified that in order to be protective, *C. neoformans* Δ*sgl1* must display on its surface certain antigenic capsular components, such as glucuronoxylomannan (GXM), because a mutant strain accumulating SGs but lacking the capsule (Δ*cap59*Δ*sgl1*) was no longer protective^36^, suggesting that SGs may act as an adjuvant to GXM.

In this study, we present the immune mechanisms by which SGs and GXM provide complete host protection in condition of CD4^+^ T cell deficiency. We found that the number of IFNγ- and IL-17A-producing T cells in the lungs were significantly increased in *C. neoformans* Δ*sgl1*-vaccinated mice compared to unvaccinated mice, even when CD4^+^ (or CD8^+^) T cells were depleted. Vaccine protection was lost when either IFNγ or IL-17A was neutralized, suggesting an innate immune cell population, such as γδ T cells, may produce these cytokines responsible for early host protection. Interestingly, mice lacking γδ T cells completely lost vaccine protection to the WT challenge, mimicking the phenotype observed during IFNγ and IL-17A neutralization. Importantly, γδ T cells robustly produced IFNγ and IL-17A when incubated with *C. neoformans* Δ*sgl1* ex vivo compared to either the WT or Δ*sgl1*Δ*cap59* strains. Because capsular GXM has been reported to bind to toll-like receptors (TLRs), we investigated the effect of the vaccination strategy in a TLR2^−/−^ mouse model. We found that γδ T cells isolated from TLR2^−/−^ mice no longer produced IFNγ or IL-17A in response to *C. neoformans* Δ*sgl1* ex vivo or in vivo, and TLR2^−/−^ mice are no longer protected from the WT strain by *C. neoformans* Δ*sgl1* vaccination.

In conclusion, our results suggest that *C. neoformans* Δ*sgl1* stimulates γδ T cells to produced IFNγ and IL-17A through a host-protective interaction between TLR2 and GXM/SGs, an immune mechanism that is still preserved in condition of CD4^+^ T cell deficiency.

## Results

### Vaccination with *C. neoformans* Δsgl1 promotes robust IFNγ- and IL-17A-expressing CD4^+^ and CD8^+^ T cells

We have recently shown that vaccination with *C. neoformans* Δ*sgl1* required either CD4^+^ or CD8^+^ T cells for complete host protection from the WT strain^41^. We therefore hypothesized that vaccination with *C. neoformans* Δ*sgl1* must induce protectively polarized T cells even in condition of CD4^+^ or CD8^+^ T cell deficiency. To test this hypothesis, mice were administered either isotype, anti-CD4, or anti-CD8 antibodies, and the number of effector cytokine-producing T cells was quantified at various timepoints during immunocompetency, CD8-deficiency, and CD4-deficiency (experimental design scheme: Supplementary Fig. 1; flow cytometry gating strategy: Supplementary Fig. 2).

**Fig. 1:**
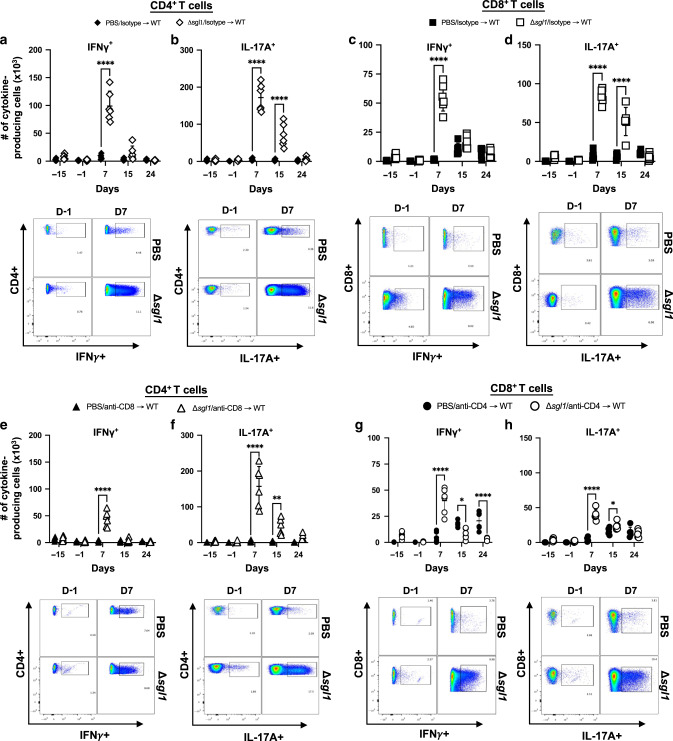
Vaccination with *C. neoformans* Δ*sgl1* drives T cell polarization towards a protective type 1/17 response in immunocompetent, CD4-deficient, and CD8-deficient mice. CBA/J mice (*n* = 6 mice/group/timepoint) were administered isotype (**a**–**d**), anti-CD8 (**e**, **f**), or anti-CD4 (**g**, **h**) antibodies. Mice then received either *C. neoformans* Δ*sgl1* (white symbols) or PBS (black symbols). After 30 days, mice were challenged with *C. neoformans* WT (day 0). On days −15, −1, 7, 15, and 24, mice were assessed for T cell-derived cytokines (IFNγ, IL-17A) via intracellular cytokine staining. Graphed data represents the mean ± SD from 2 independent experiments (*n* = 3 mice/group/timepoint for each biological replicate). Representative flow cytometry plots for days −1 and 7 for each group are shown, which were gated from live, CD45^+^ singlets (gating scheme shown in Supplementary Fig. 2). Significance was determined by a two-way ANOVA using Šídák’s multiple comparisons test for *P* value adjustment, and significance is denoted as *P < 0.05; **P < 0.01; ***P < 0.005; ****P < 0.001.

*C. neoformans* Δ*sgl1*-vaccinated and unvaccinated immunocompetent mice (isotype antibody treated) were first assessed for T cell cytokine responses in both CD4^+^ (Fig. 1a, b) and CD8^+^ (Fig. 1c, d) subsets. Primarily, we observed that vaccinated mice had a significantly greater number of IFNγ- (Fig. 1a, c) and IL-17A-producing (Fig. 1b, d) T cells than unvaccinated mice, particularly on day 7 post WT challenge, that subsided to baseline levels by day 15 or 24 post challenge. We also observed that the number of IL-13-producing T cells in *C. neoformans* Δ*sgl1*-vaccinated mice was less than or equal to that of unvaccinated mice, particularly post WT challenge (Supplementary Fig. 3A, B). These results suggest that vaccination with *C. neoformans* Δ*sgl1* drives robust CD4^+^ and CD8^+^ T cell responses producing IFNγ and IL-17A in response to the WT challenge.

We next examined the T cell cytokine expression profile of vaccinated and unvaccinated mice during immunodeficiency by quantifying the number of effector cytokine-producing CD4^+^ T cells in CD8-depleted mice (Fig. 1e, f) and the number of effector cytokine-producing CD8^+^ T cells in CD4-depleted mice (Fig. 1g, h). In CD8-depleted *C. neoformans* Δ*sgl1*-vaccinated mice, there was an earlier and significantly greater number of IFNγ- (Fig. 1e) and IL-17A-producing (Fig. 1f) CD4^+^ T cells compared to unvaccinated mice on day 7 post challenge, which both subsided by day 15. However, the number of IL-13-producing CD4^+^ T cells was significantly greater in unvaccinated mice compared to vaccinated mice at all timepoints post WT challenge (Supplementary Fig. 3C).

In CD4-depleted *C. neoformans* Δ*sgl1*-vaccinated mice, there was also an earlier and significantly greater number of IFNγ- (Fig. 1g) and IL-17A-producing (Fig. 1h) CD8^+^ T cells compared to unvaccinated mice on day 7 post WT challenge, which both returned to baseline levels by day 15 or 24. Although unvaccinated mice had a greater number of IL-13-producing CD8^+^ T cells on day 7 post WT challenge, the number of IL-13-producing CD8^+^ T cells in vaccinated mice were significantly greater on days 15 and 24 post challenge (Supplementary Fig. 3D).

Interestingly, the magnitude of the IFNγ- and IL-17A-producing CD4^+^ T cell response was noticeably greater than that of the IFNγ- and IL-17A-producing CD8^+^ T cell response (Fig. 1a–d). This same trend was also apparent for the IFNγ- and IL-17A-producing CD4^+^ T cell response during CD8-deficiency compared to the IFNγ- and IL-17A-producing CD8^+^ T cell response during CD4-deficiency (Fig. 1e–h), although this difference may possibly be attributed to experimental variation between different groups of mice. Taken together, these data suggest that vaccination with *C. neoformans* Δ*sgl1* strongly promotes protective CD4^+^ or CD8^+^ T cell responses in terms of both the effector cytokine secretion profile and the response time to a WT challenge during immunocompetency, CD8-deficiency, and CD4-deficiency.

### Both IFNγ and IL-17A are required for *C. neoformans* Δsgl1-induced host protection

Because we found that both CD4^+^ and CD8^+^ T cells were potent sources of IFNγ and IL-17A post WT challenge during immunocompetency, CD8-deficiency, and CD4-deficiency in *C. neoformans* Δ*sgl1*-vaccinated mice, we asked if these cytokines themselves were essential for host protection in *C. neoformans* Δ*sgl1*-vaccinated mice.

**Fig. 2:**
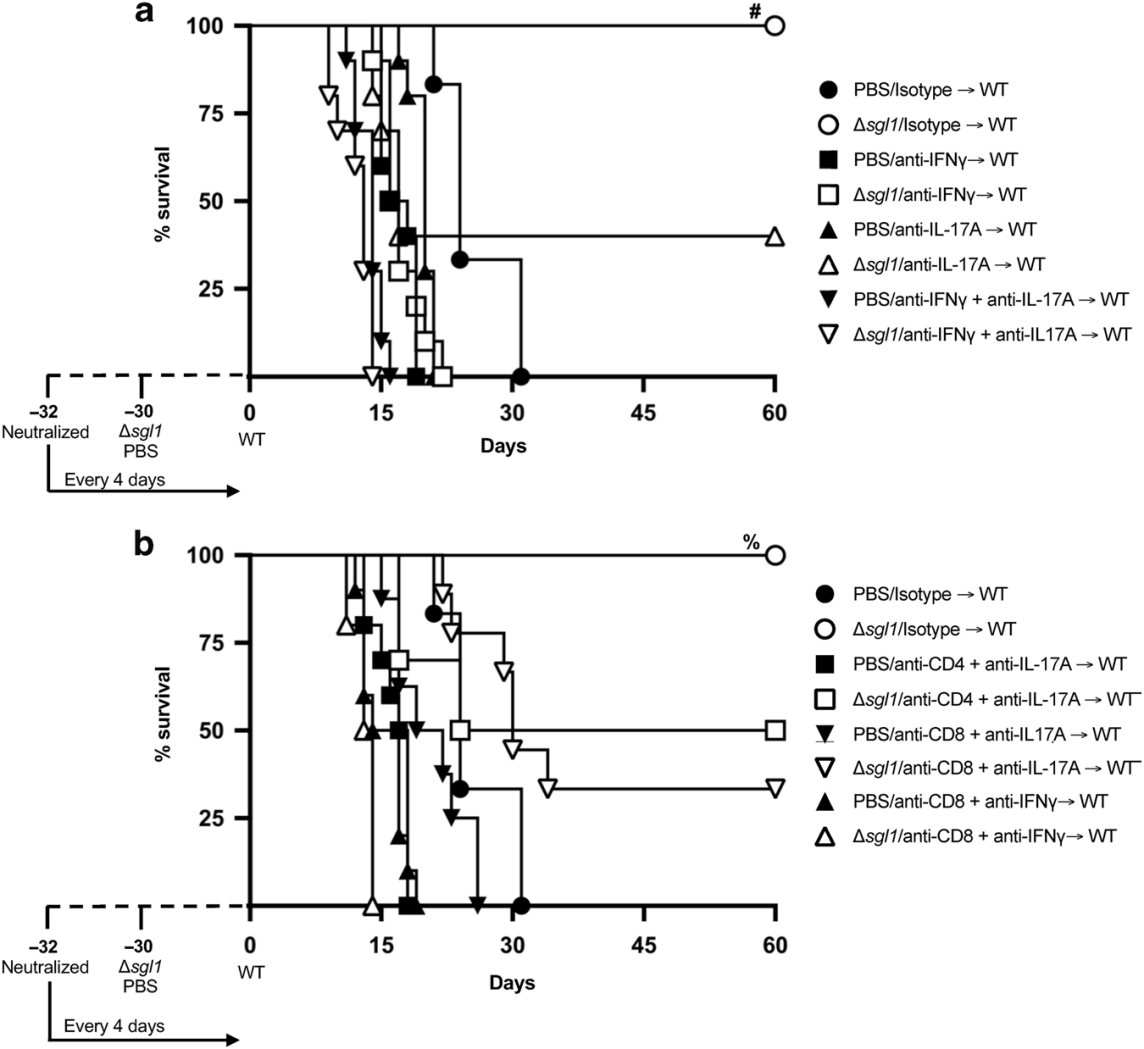
Host protection from *C. neoformans* Δ*sgl1* vaccination is lost in the absence of IFNγ or IL-17A. CBA/J mice (*n* = 8–10 mice/group) were neutralized of specific cytokines and/or cell types (as indicated in the figure legends) prior to vaccination with *C. neoformans* Δ*sgl1* (white symbols) or PBS controls (black symbols), and neutralizations continued for the entirety of the experiment at noted intervals. **a**, **b**. Vaccinated and unvaccinated mice were challenged with *C. neoformans* WT strain and assessed for survival during specific cytokine neutralization (**a**) or cytokine neutralization during either CD4 or CD8 T cell deficiency (**b**). Survival significance was determined by the Mantel–Cox log-rank test, and denoted on each graph: **a**. ^#^*P* < 0.001 for Δ*sgl1*/Isotype → WT vs. Δ*sgl1*/anti-IL-17A → WT; **b**. ^%^*P* < 0.001 for Δ*sgl1*/Isotype → WT vs. Δ*sgl1*/anti-CD4 + anti-IL-17A → WT or Δ*sgl1*/anti-CD8 + anti-IL-17A → WT.

Mice were neutralized of either IFNγ or IL-17A via intraperitoneal administration of monoclonal antibodies specific for each respective cytokine prior to vaccination and continued for the entirety of the experiment to maintain their absence. We first investigated mouse survival during administration of *C. neoformans* Δ*sgl1* and found that all mice depleted of both CD4^+^ T cells and IFNγ fatally succumbed to *C. neoformans* Δ*sgl1* (Supplementary Fig. 4A). Thus, it was not possible to subsequently test the effect of WT challenge under this condition. For the other cytokine neutralization conditions, we found that all vaccinated and unvaccinated mice neutralized of IFNγ fully succumbed to WT challenge within 20 days in either the presence (Fig. 2a) or absence (Fig. 2b) of CD4^+^ or CD8^+^ T cells. Additionally, while all unvaccinated mice fully succumbed to infection in the absence of IL-17A, vaccinated mice neutralized of IL-17A in either the presence (Fig. 2a) or absence (Fig. 2b) of CD4^+^ or CD8^+^ T cells exhibited a ~35% survival rate 60 days post WT challenge. However, endpoint organ fungal burden analysis confirmed that the surviving vaccinated mice had profound extrapulmonary fungal dissemination to the brain, spleen, liver, and kidneys indicating a lack of lung containment in the absence of IL-17A (Supplementary Fig. 4B, C). Overall, these data suggest that both IFNγ and IL-17A were required for complete host protection and pulmonary containment of the WT strain in *C. neoformans* Δ*sgl1-*vaccinated mice regardless of T cell immunodeficiency.

Because we observed a ~35% survival rate for vaccinated mice neutralized of IL-17A, we wanted to further assess the necessity of IL-17A via a WT challenge in *C. neoformans* Δ*sgl1-*vaccinated mice depleted of neutrophils, the main effector cell working in an IL-17-directed mechanism. In concert with the IL-17A neutralization experiments, vaccinated mice depleted of neutrophils partially succumbed to the WT challenge resulting in a 30% survival rate (Supplementary Fig. 4D) closely mirroring host survival when IL-17A was neutralized (Fig. 2a, b). Therefore, these data suggest that neutrophils functioning through IL-17A provide essential host protection upon WT challenge in *C. neoformans* Δ*sgl1*-vaccinated mice.

### *C. neoformans* Δsgl1-vaccinated mice exhibit a robust γδ T cell response

Since both IFNγ and IL-17A were found to be independently required for complete host protection in *C. neoformans* Δ*sgl1*-vaccinated mice (Fig. 2a, b), we hypothesized that there must be other significant cellular sources of these host-protective cytokines working earlier on in the lungs prior to the adaptive CD4^+^ and/or CD8^+^ T cell responses. The hypothesized cell type must (i) not be lost during CD4^+^ and/or CD8^+^ T cell depletion conditions, (ii) not be lost during any prior depletion condition in our previous study regarding the avirulence of *C. neoformans* Δ*sgl1* during immunocompromised conditions^41^, and (iii) be a potent innate source of IFNγ and/or IL-17A.

**Fig. 3:**
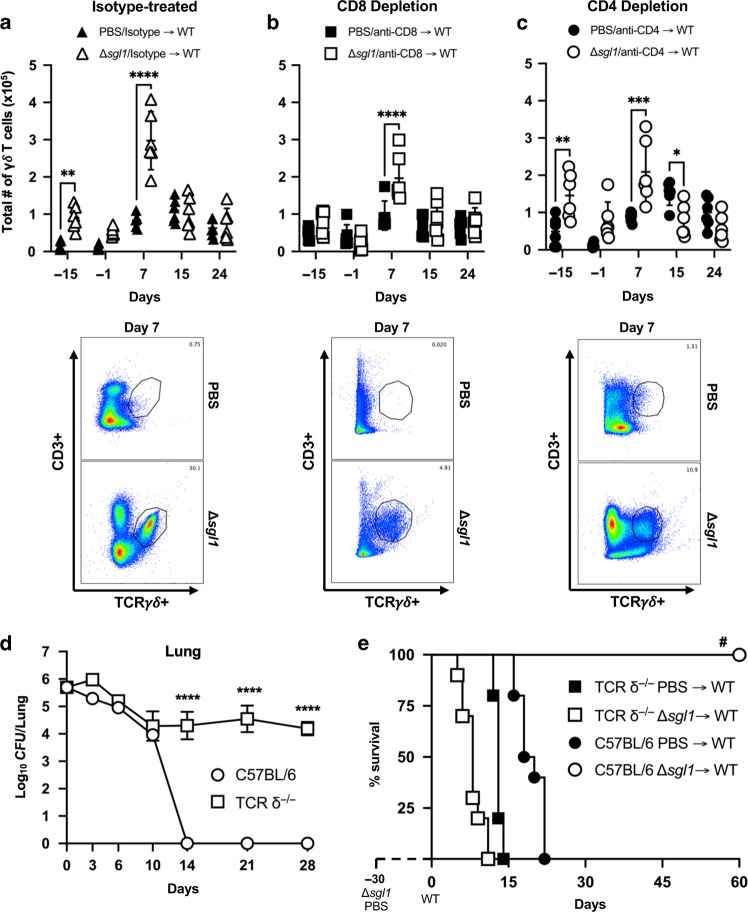
γδ T cells are required for the clearance of *C. neoformans* Δ*sgl1* and elicited host vaccine protection. CBA/J mice (*n* = 6 mice/group/timepoint) were administered isotype (**a**), anti-CD8 (**b**), or anti-CD4 (**c**) antibodies then received either *C. neoformans* Δ*sgl1* (white symbols) or PBS (black symbols). After 30 days, mice were challenged with *C. neoformans* WT (day 0). On days −15, −1, 7, 15, and 24, the number of γδ T cells in the lungs were quantified via flow cytometry. **d**. Lung fungal burden was assessed in C57BL/6 or TCRδ^−/−^ mice at set timepoints during inoculation with *C. neoformans* Δ*sgl1* (*n* = 3–6 mice/group/timepoint). **e**. Survival of *C. neoformans* Δ*sgl1*-vaccinated or unvaccinated (PBS controls) C57BL/6 or TCRẟ^−/−^ mice upon lethal WT challenge (*n* = 10 mice/group). Graphed data represents the mean ± SD from 2 independent experiments (*n* = 3 mice/group/timepoint for each biological replicate) (**a**–**c**) or survival percentage of challenged mice (**e**). Representative flow cytometry plots for day 7 for each group are shown, which were gated from live, CD45^+^ singlets (gating scheme shown in Supplementary Fig. 5). Significance was determined by a two-way ANOVA using Šídák’s multiple comparisons test for *P* value adjustment (**a–d**), and significance is denoted as *P < 0.05; **P < 0.01; ***P < 0.005; ****P < 0.001. The Mantel-Cox log-rank test was used to determine survival significance on graph **e**: #, *P* < 0.001 for C57BL/6 Δ*sgl1* → WT vs. TCRẟ^−/−^ Δ*sgl1* → WT.

Abiding by these conditions, γδ T cells were investigated since they are an innate-like lymphocyte population that have been well-documented to produce IFNγ and IL-17A in response to antigen encounter at mucosal barrier tissues, such as the intestines, skin, and lungs^43–45^, and were not targeted by any of the antibody depletions carried out in our prior study^41^. Thus, we examined the recruitment of γδ T cells to the lungs via flow cytometry (flow cytometry gating strategy: Supplementary Fig. 5) in *C. neoformans* Δ*sgl1*-vaccinated and unvaccinated mice during immunocompetent (Fig. 3a), CD8-deficient (Fig. 3b), or CD4-deficient (Fig. 3c) conditions both pre and post WT challenge. Primarily, all immunocompetent, CD8-deficient, and CD4-deficient mice administered *C. neoformans* Δ*sgl1* displayed significantly greater numbers of γδ T cells on day −15 compared to unvaccinated mice indicating that γδ T cells actively responded to *C. neoformans* Δ*sgl1*. Additionally, significantly greater numbers of γδ T cells were observed for all *C. neoformans* Δ*sgl1*-vaccinated mice regardless of depletion status on day 7 post WT challenge as well (Fig. 3a–c), and the numbers of γδ T cells all subsided to baseline levels by day 15 post WT challenge. Overall, these data suggest that vaccination with *C. neoformans* Δ*sgl1* stimulates a robust recruitment of γδ T cells to the lungs independent of CD4^+^ or CD8^+^ T cells during the vaccine administration stage and more importantly post WT challenge.

### γδ T cells are essential for host protection in *C. neoformans* Δ*sgl1*-vaccinated mice

Since we observed a robust γδ T cell response during both administration of *C. neoformans* Δ*sgl1* and post WT challenge in vaccinated mice, we then investigated whether γδ T cells were required for host protection using TCRδ^−/−^ mice genetically lacking γδ T cells. We first found that TCRδ^−/−^ mice were unable to clear *C. neoformans* Δ*sgl1* from the lungs by day 28 post administration (Fig. 3d). In addition, significantly increased extrapulmonary dissemination of the mutant was observed in the brain, spleen, liver, and kidneys beginning as early as day 3 post administration (Supplementary Fig. 6). Because these mice had not exhibited any mortality to the mutant and fungal clearance was previously shown to not be necessary for host protection^41^, *C. neoformans* Δ*sgl1*-vaccinated TCRδ^−/−^ mice were subsequently challenged with the WT strain and assessed for survival. All vaccinated TCRδ^−/−^ mice fatally succumbed to the WT infection at a similar rate to unvaccinated TCRδ^−/−^ mice (Fig. 3e). These data suggest that γδ T cells are essential for both initial host clearance and containment of *C. neoformans* Δ*sgl1* as well as protection of *C. neoformans* Δ*sgl1*-vaccinated mice against a lethal WT challenge.

### γδ T cells produce both IFNγ and IL-17A in response to live or HK *C. neoformans* Δsgl1 ex vivo

Since γδ T cells were found to be required for both host control of *C. neoformans* Δ*sgl1* and vaccine protection from the WT strain, we investigated whether γδ T cells directly produced IFNγ or IL-17A in response to *C. neoformans* Δ*sgl1*. To test this, spleens were harvested from uninfected TCRβ^−/−^ mice, which genetically lack CD4^+^ and CD8^+^ T cells and consist mainly of γδ T cells, and these splenocytes were cultured ex vivo with plate-bound anti-TCRγδ. Due to the small number of circulating or tissue-resident γδ T cells in uninfected, naive mice, the spleens were the greatest source of γδ T cells for both number and origin for lung-recruited γδ T cells post infection. The cultured splenocytes were stimulated with the following fungal strains: (i) *C. neoformans* WT, which produces capsular GXM but does not accumulate SGs; (ii) *C. neoformans* Δ*sgl1*, which produces GXM and accumulates SGs; or (iii) *C. neoformans* Δ*cap59*Δ*sgl1*, which is an acapsular strain that does not produce GXM but accumulates SGs. Supernatants were collected on days 1, 3, and 5 post stimulation and examined for IFNγ and IL-17A production via ELISA.

**Fig. 4:**
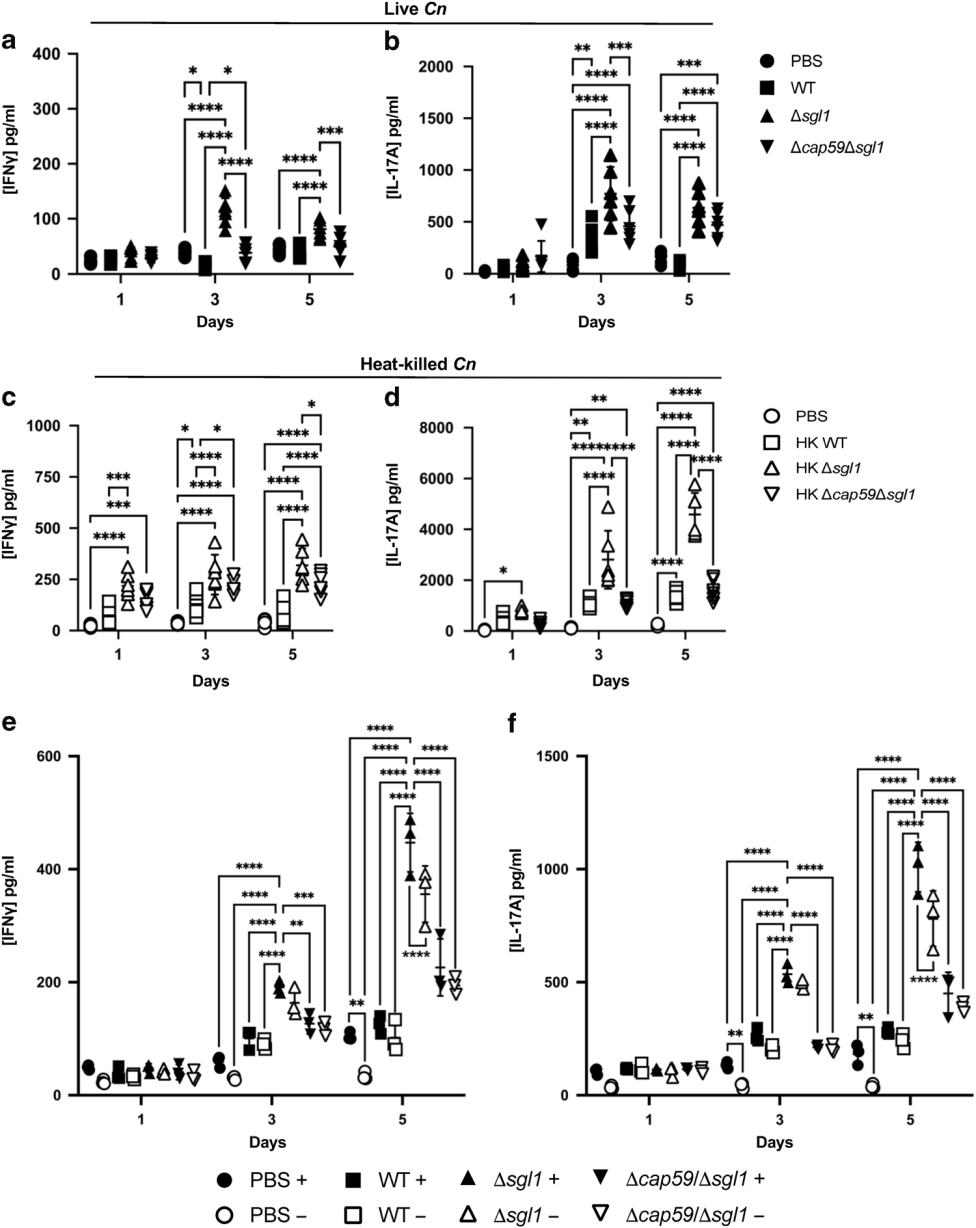
γδ T cells respond to *C. neoformans* (*Cn*) Δ*sgl1* via the production of IFNγ and IL-17A. **a**–**d**. Splenocytes from uninfected TCRβ^−/−^ mice were processed and cultured ex vivo with plate-bound anti-TCRγδ. These cultured cells were stimulated with PBS, live *C. neoformans* WT, *C. neoformans* Δ*sgl1*, or *C. neoformans* Δ*cap59*Δ*sgl1* (**a**, **b**) or PBS, heat-killed (HK) *C. neoformans* WT, HK *C. neoformans* Δ*sgl1*, or HK *C. neoformans* Δ*cap59*Δ*sgl1* (**c**, **d**). On days 1, 3, and 5 post stimulation, supernatants were collected and assessed for production of IFNγ (**a**, **c**) or IL-17A (**b**, **d**) via ELISA. **e**, **f**. γδ T cells were purified from the spleens of uninfected C57BL/6 mice via MACS separation kit and cultured ex vivo with (+) or without (−) plate-bound anti-TCRγδ, stimulated with PBS, live *C. neoformans* WT, *C. neoformans* Δ*sgl1*, or *C. neoformans* Δ*cap59*Δ*sgl1*, and assessed for IFNγ (**e**) and IL-17A (**f**) production on days 1, 3, and 5 post stimulation. Graphed data represents the mean ± SD and are representative of 2 independent experiments (*n* = 3 mice/group/timepoint for each biological replicate) (**a**–**d**) or a single replicate of *n* = 3 mice/group/timepoint (**e**, **f**). Significance was determined by a two-way ANOVA using Šídák’s multiple comparisons test for *P* value adjustment, and significance is denoted as *P < 0.05; **P < 0.01; ***P < 0.005; ****P < 0.001.

We found a significant increase in IFNγ production when splenocytes were stimulated with *C. neoformans* Δ*sgl1* compared to either the WT or *C. neoformans* Δ*cap59*Δ*sgl1* strains on day 3 and to the WT strain on day 5 post stimulation (Fig. 4a). Similarly, significantly greater production of IL-17A was observed during stimulation with *C. neoformans* Δ*sgl1* compared to the WT strain on days 3 and 5 post stimulation (Fig. 4b). These data suggest that splenocytes from TCRβ^−/−^ mice, mainly consisting of γδ T cells, recognize and functionally respond better to *C. neoformans* Δ*sgl1* compared to the WT or *C. neoformans* Δ*cap59*Δ*sgl1* via the production of both IFNγ and IL-17A.

*C. neoformans* Δ*sgl1* has indeed been shown to induce robust host immunity^32,36,41^, but a live, attenuated vaccine strain does not provide sufficient clinical application. We have recently shown that heat-killed (HK) *C. neoformans* Δ*sgl1* provided full host protection during immunocompetency or immunodeficiency^42^, so we asked if stimulation with HK *C. neoformans* Δ*sgl1* would also result in the significant production of these protective cytokines. Utilizing the same experimental procedure performed with the live strain stimulation of murine splenocytes, we cultured splenocytes from TCRβ^−/−^ mice ex vivo with identical HK preparations of the same three strains and quantified the resultant production of IFNγ and IL-17A. Very interestingly, we found that stimulation with HK *C. neoformans* Δ*sgl1* exhibited significantly greater production of IFNγ compared to the HK WT strain on day 5 post stimulation (Fig. 4c) and significantly greater production of IL-17A compared to both the HK WT strain and HK *C. neoformans* Δ*cap59*Δ*sgl1* on days 3 and 5 post stimulation (Fig. 4d). Of noteworthiness, the magnitude of cytokine production from TCRβ^−/−^ splenocytes was ~3-fold greater for both IFNγ and IL-17A from stimulation with the HK strains compared to the live strains (Fig. 4a–d). Taken together, these data suggest that TCRβ^−/−^ splenocytes responded to both live and HK *C. neoformans* Δ*sgl1* stimulation ex vivo with significantly greater production of both IFNγ and IL-17A compared to the non-protective WT or *C. neoformans* Δ*cap59*Δ*sgl1* strains.

### TCRγδ crosslink is not necessary for the protective cytokine production from ex vivo stimulation with *C. neoformans* Δsgl1

We have uncovered that TCRβ^−/−^ splenocytes predominately made up of by γδ T cells produced significantly increased concentrations of IFNγ and IL-17A, but we wanted to know if (i) this response was due to the heterogeneous population of leukocytes within the spleen and (ii) if the crosslink of the γδTCR via the plate-bound antibody was the driving force of the observed response. To answer these questions, we purified γδ T cells from uninfected TCRβ^−/−^ spleens and cultured these purified cells with or without plate-bound anti-TCRγδ antibody with stimulation by the same three strains used previously.

Similarly to the crude spleen preparations, we found that stimulation of γδ T cells with *C. neoformans* Δ*sgl1* produced significantly greater concentrations of IFNγ (Fig. 4e) and IL-17A (Fig. 4f) on days 3 and 5 compared to stimulation with any other condition. Very interestingly, we did not observe a statistical difference in IFNγ or IL-17A production between the presence or the absence of plate-bound anti-TCRγδ antibody for any stimulation conditions on day 3. Differences in cytokine production were observed within the stimulation conditions with or without plate-bound antibody on day 5 post stimulation for the control PBS-stimulated cells and *C. neoformans* Δ*sgl1*-stimulated cells (Fig. 4e, f). Moreover, this same trend was observed for purified γδ T cells with HK *C. neoformans* Δ*sgl1* compared to the other HK strains, where the presence or absence did not influence day 3 production differences but was observed on day 5 (Supplementary Fig. 7A, B). Taken together, these data suggest that γδ T cells can directly respond to *C. neoformans* Δ*sgl1* ex vivo with robust production of IFNγ and IL-17A without the need for crosslink of the γδTCR.

### γδ T cells require TLR2 for the production of IFNγ and IL-17A in response to *C. neoformans* Δsgl1

Since we have uncovered that γδ T cells can directly respond to *C. neoformans* Δ*sgl1* without the need for TCR crosslinking, we then sought to determine the mechanism by which γδ T cells recognized *C. neoformans* Δ*sgl1* through the exploration of host pattern recognition receptors (PRRs). To our knowledge, there are no reports in the literature of PRRs interacting with fungal-derived SGs. However, there are numerous studies in the literature reporting that GXM from virulent strains, such as the WT *C. neoformans* strain H99 used in this study, displayed immunosuppressive properties by dampening the pro-inflammatory host immune response of phagocytes and T lymphocytes. With regards to this, toll-like receptor (TLR) 2 and TLR4 are host PRRs that have been shown to exhibit attenuated responses due to interaction with GXM but are also known to be host PRRs responsible for the production of pro-inflammatory cytokines from γδ T cells^44,46–50^. In particular, TLR2 ligation primarily leads to the production of IL-17A from γδ T cells, so TLR2 was chosen to be investigated as a potential mechanism for the host-protective cytokine production upon administration of *C. neoformans* Δ*sgl1*.

**Fig. 5:**
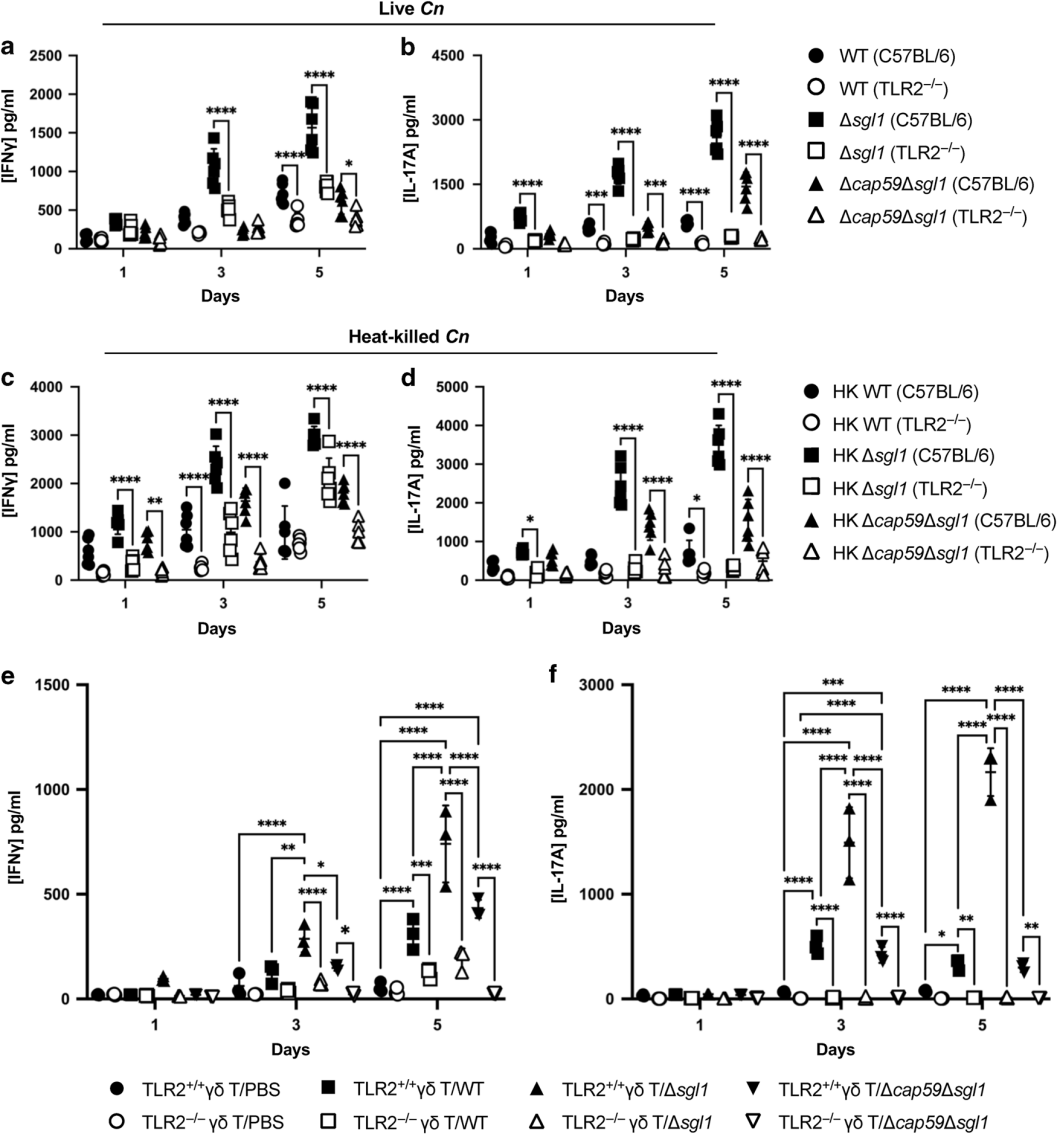
Role of toll-like receptor 2 (TLR2) on *C. neoformans* (*Cn*) Δ*sgl1*-induced cytokine production by γδ T cells ex vivo. **a**–**d**. γδ T cells were purified from the spleens of uninfected C57BL/6 or TLR2^−/−^ mice via MACS separation kit and cultured ex vivo with plate-bound anti-TCRγδ. These cultured cells were stimulated with live *C. neoformans* WT, *C. neoformans* Δ*sgl1*, or *C. neoformans* Δ*cap59*Δ*sgl1* (**a**, **b**) or heat-killed (HK) *C. neoformans* WT, HK *C. neoformans* Δ*sgl1*, or HK *C. neoformans* Δ*cap59*Δ*sgl1* (**c**, **d**). On days 1, 3, and 5 post stimulation, supernatants were collected and assessed for production of IFNγ (**a**, **c**) or IL-17A (**b**, **d**) via ELISA. **e**, **f**. γδ T cells were purified from the spleens of uninfected C57BL/6 (TLR2^+/+)^ or TLR2^−/−^ mice via MACS separation kit and co-cultured ex vivo with plate-bound anti-TCRγδ. TLR2^+/+^ γδ T cells were cultured with TLR2^−/−^ antigen presenting cells (APCs) or TLR2^−/−^ γδ T cells were cultured with TLR2^+/+^ APCs, stimulated with PBS, live *C. neoformans* WT, *C. neoformans* Δ*sgl1*, or *C. neoformans* Δ*cap59*Δ*sgl1*, and assessed for IFNγ (**e**) and IL-17A (**f**) on days 1, 3 and 5 post stimulation. Graphed data represents the mean ± SD and are representative of 2 independent experiments (*n* = 3 mice/group/timepoint for each biological replicate) (**a**–**d**) or a single replicate of *n* = 3 mice/group/timepoint (**e**, **f**). Significance was determined by a two-way ANOVA using Šídák’s multiple comparisons test for *P* value adjustment, and denoted as *P < 0.05; **P < 0.01; ***P < 0.005; ****P < 0.001.

To test the necessity of TLR2 on γδ T cells for the recognition of *C. neoformans* Δ*sgl1* and subsequent production of IFNγ and IL-17A, we utilized two ex vivo approaches. First, we purified γδ T cells from the spleens of C57BL/6 and TLR2^−/−^ mice, cultured them ex vivo with plate-bound anti-TCRγδ, and stimulated them with live or HK *C. neoformans* WT, *C. neoformans* Δ*sgl1*, or *C. neoformans* Δ*cap59*Δ*sgl1* (Fig. 5a–d). Intriguingly, there was a significantly attenuated IFNγ response in TLR2^−/−^ stimulated γδ T cells compared to the γδ T cells from C57BL/6 for both live strain stimulation on days 3 and 5 post stimulation (Fig. 5a) and HK stimulation on days 1, 3, and 5 post stimulation (Fig. 5c), although the levels of IFNγ still displayed an increasing trend from day 1 to day 5 post stimulation with live or HK *C. neoformans* Δ*sgl1*. On the other hand, there was a significant difference between the IL-17A response in TLR2^−/−^ stimulated γδ T cells compared to the γδ T cells from C57BL/6 for both live strain stimulation on days 1, 3, and 5 post stimulation (Fig. 5b) and HK stimulation on days 3 and 5 post stimulation (Fig. 5d). However, unlike the IFNγ response, the measured IL-17A response was completely ablated in TLR2^−/−^ γδ T cells during all timepoints and stimulation conditions including *C. neoformans* Δ*sgl1*. These data clearly suggest that TLR2 plays a contributing role in IFNγ production and a vital role in IL-17A production by murine γδ T cells in the recognition and host-protective response to SGs and GXM from *C. neoformans* Δ*sgl1*.

Second, we utilized a co-culture approach to determine the necessity of TLR2 on γδ T cells compared to other antigen presenting cells (APCs) for the observed protective host cytokine response. As an intermediate cell population in our γδ T cell purification protocol, we exclude CD11b^+^ leukocytes made up of APCs. We then cultured CD11b^+^ APCs from C57BL/6 mice (TLR2^+/+^ APCs) with TLR2^−/−^ γδ T cells and vice versa (TLR2^−/−^ APCs with TLR2^+/+^ γδ T cells) with all stimulation conditions for the production of IFNγ and IL-17A (Fig. 5e, f and Supplementary Fig. 8). Similarly to the purified γδ T cell experiments above, we observed an attenuated IFNγ response only when TLR2^−/−^ γδ T cells were cultured with TLR2^+/+^ APCs and stimulated with the various live or HK culture conditions (Fig. 5e and Supplementary Fig. 8A), but this was not observed for the opposite conditions (TLR2^+/+^ γδ T cells with TLR2^−/−^ APCs) where *C. neoformans* Δ*sgl1* elicited the greatest cytokine response. The same was observed for the production of IL-17A where TLR2 was only necessary on the γδ T cells but not the APCs (Fig. 5f and Supplementary Fig. 8B). In all cases, there was a normal rate of production of IFNγ or IL-17A when stimulated with *C. neoformans* Δ*sgl1* in conditions of TLR2^+/+^ γδ T cells even though TLR2 was absent on the co-cultured APCs. Taken together, TLR2 is required on γδ T cells for the observed production of IFNγ and IL-17A in response to live or HK *C. neoformans* Δ*sgl1*.

### γδ T cells are the main producers of IL-17A in response to *C. neoformans* Δsgl1 and WT challenge

**Fig. 6:**
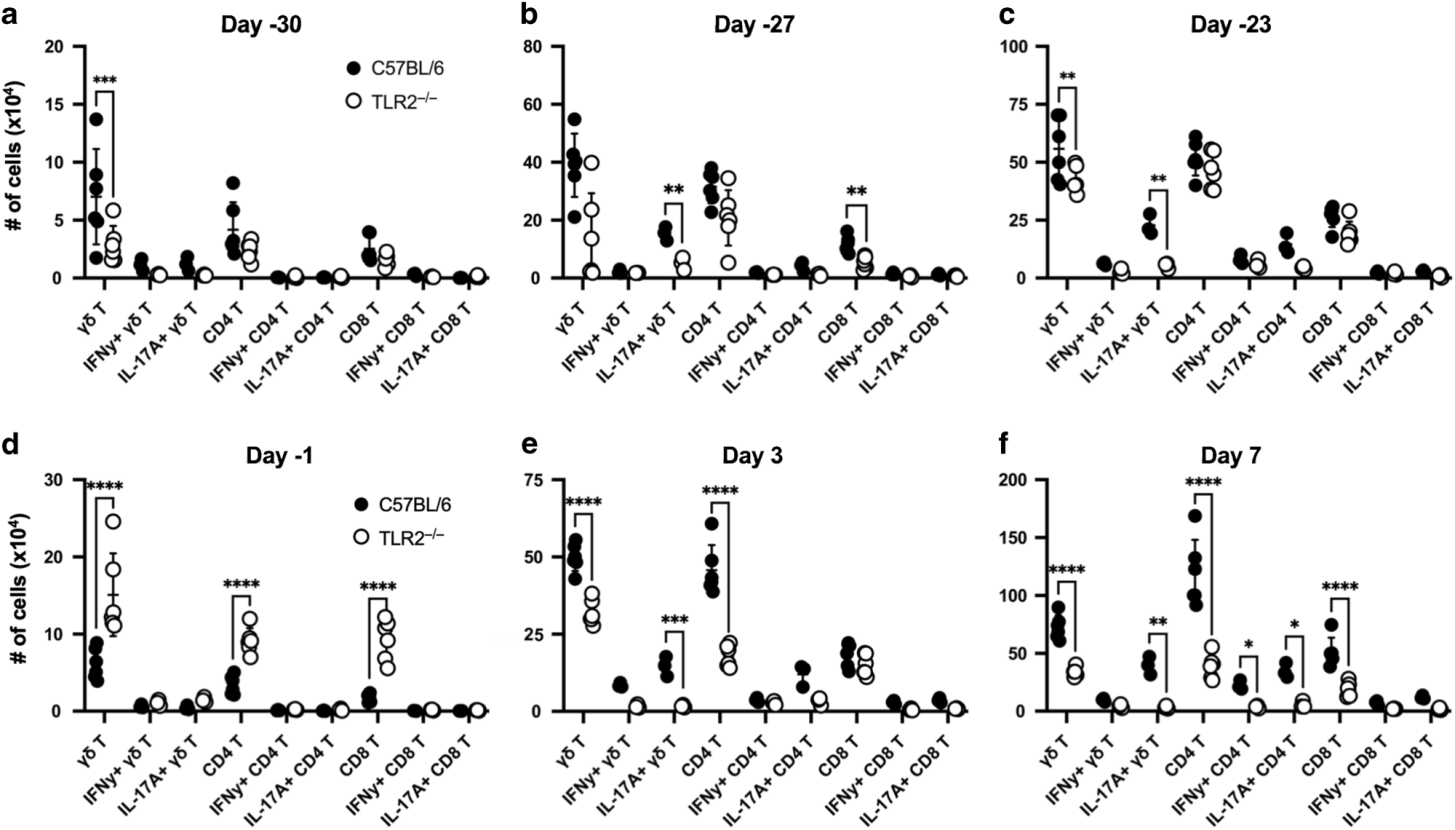
Toll-like receptor 2 (TLR2) is required in vivo for protective cytokine-producing γδ T cells to *C. neoformans* Δ*sgl1*. C57BL/6 (black symbols) or TLR2^−/−^ (white symbols) were administered *C. neoformans* Δ*sgl1* and assessed for T cell-derived cytokines via intracellular cytokine stimulation on days −30 (uninfected; **a**), −27 (**b**), and −23 (**c**). 30 days after *C. neoformans* Δ*sgl1* administration, mice were challenged with *C. neoformans* WT and assessed for T cell-derived cytokines via intracellular cytokine stimulation on days −1 (unchallenged; **d**), 3 (**e**), and 7 (**f**). At all timepoints, mice were assessed for the total number of γδ T, CD4^+^, and CD8^+^ T cells as well as the number of IFNγ- or IL-17A-producing subsets of these cells. Graphed data represent the mean ± SD and are representative of 1–2 independent experiments (*n* = 3–6 mice/timepoint/group). Significance was determined by a two-way ANOVA using Šídák’s multiple comparisons test for *P* value adjustment, and denoted as *P < 0.05, **P < 0.01, ***P < 0.005, ****P < 0.001.

Because we have now uncovered a potential mechanistic role for TLR2 in the host-protective response to *C. neoformans* Δ*sgl1* ex vivo, we then investigated the role of TLR2 on host vaccine protection by *C. neoformans* Δ*sgl1* to WT challenge in vivo. We utilized intracellular cytokine staining in C57BL/6 and TLR2^−/−^ mice both early on post *C. neoformans* Δ*sgl1* administration (Fig. 6a–c) and early on post WT challenge (Fig. 6d–f). Compared to uninfected mice, there was an observable increase in the total number of γδ, CD4^+^, and CD8^+^ T cells 3 days post *C. neoformans* Δ*sgl1* administration (Day −27; Fig. 6a, b). However, although the total number of γδ T cells was not different between C57BL/6 and TLR2^−/−^ mice, the number of IL-17A-producing γδ T cells was significantly greater in C57BL/6 compared to TLR2^−/−^ mice, while there were no differences in the numbers of cytokine-producing CD4^+^ or CD8^+^ T cells (Fig. 6b). Furthermore, by day 7 post *C. neoformans* Δ*sgl1* administration (Day −23), there is a continual increase in the number of γδ, CD4^+^, and CD8^+^ T cells although γδ T cells remain as the dominant population of IL-17A-producing cells (Fig. 6c).

When we look at the WT challenge phase, we first observed that the total number of γδ, CD4^+^, and CD8^+^ T cells had decreased by ~3–5-fold in C57BL/6 mice and was significantly lower than the total number of these cells in TLR2^−/−^ mice 1 day prior to WT challenge (Fig. 6d). Nonetheless, there was no difference in the cytokine-producing T cell numbers between the two groups of mice. By days 3 and 7 post WT challenge, the total numbers of γδ, CD4^+^, and CD8^+^ T cells in C57BL/6 mice have increased significantly compared to in TLR2^−/−^ mice (Fig. 6e, f). Moreover, γδ T cells remained as the dominant IL-17A-producing cell type although CD4^+^ T cells begin to rapidly increase from day 3 to day 7. Regardless of the vaccination or challenge phases, CD8^+^ T cells were not observed as significant cytokine-producing cells in C57BL/6 compared to TLR2^−/−^ mice. Ultimately, these data suggest that γδ T cells are a major source of IL-17A production in vivo and TLR2^−/−^ mice lose the cytokine response of these cells to either *C. neoformans* Δ*sgl1* or the WT strain.

### Vaccination with *C. neoformans* Δ*sgl1* requires TLR2 for host protection

**Fig. 7:**
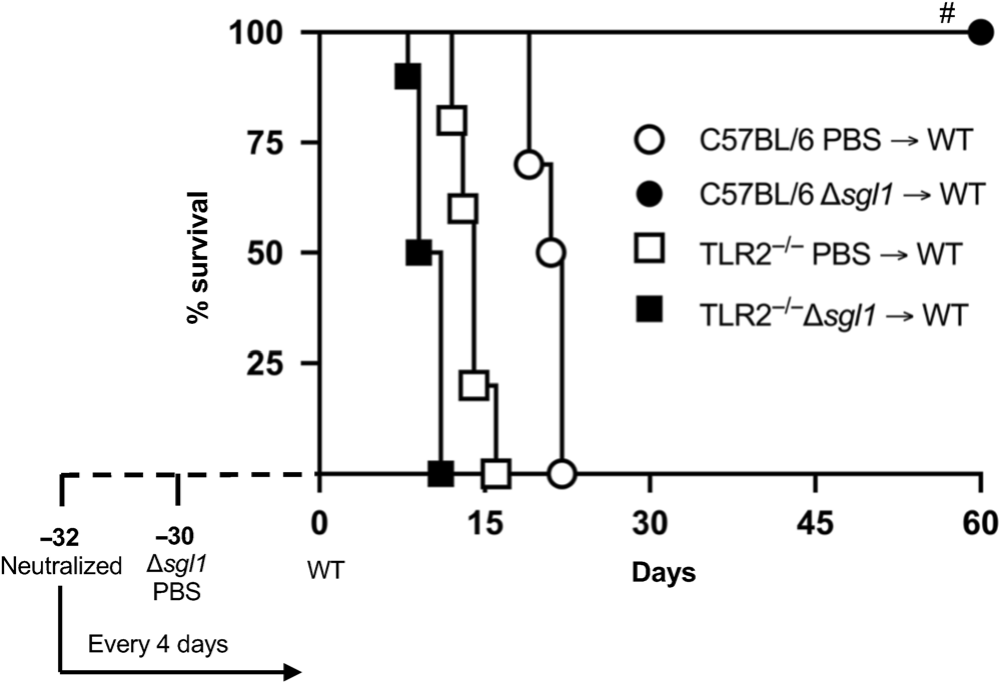
Toll-like receptor 2 (TLR2) is required for *C. neoformans* Δ*sgl1*-induced host protection. Survival of *C. neoformans* Δ*sgl1*-vaccinated or unvaccinated (PBS controls) C57BL/6 or TLR2^−/−^ mice upon lethal WT challenge (*n* = 10 mice/group). Graphed data represent the mice survival percentage. The Mantel–Cox log-rank test was used to determine survival significance: ^#^*P* < 0.001 for C57BL/6 Δ*sgl1* → WT vs. TLR2^−/−^ Δ*sgl1* → WT.

Since we found that TLR2^−/−^ mice lacked a proper IFNγ and IL-17A response in vivo, we first wanted to assess the role of TLR2 on the clearance and containment of C57BL/6 and TLR2^−/−^ mice post *C. neoformans* Δ*sgl1* administration. Both C57BL/6 and TLR2^−/−^ mice displayed 100% survival at the 30 day experimental endpoint (Supplementary Fig. 9A). However, while all C57BL/6 mice cleared *C. neoformans* Δ*sgl1* from the lungs with no observed extrapulmonary fungal dissemination, TLR2^−/−^ mice displayed a large fungal burden in the lungs as well as significant extrapulmonary dissemination to the brain, spleen, liver, and kidneys (Supplementary Fig. 9B) suggesting that TLR2 is required for host clearance and containment of our mutant to the lungs. Next, we then assessed the survival of *C. neoformans* Δ*sgl1*-vaccinated and unvaccinated C57BL/6 or TLR2^−/−^ mice against a WT challenge, and we found that vaccinated TLR2^−/−^ mice fully succumbed to fatal WT infection at a similar rate to unvaccinated TLR2^−/−^ mice (Fig. 7). Together, these data strongly suggest that host vaccination with *C. neoformans* Δ*sgl1* requires TLR2 for both control of *C. neoformans* Δ*sgl1* and protection against the WT strain.

## Discussion

In this study, we provide evidence for the underlying immune mechanism of host vaccine protection by *C. neoformans* Δ*sgl1* during the predisposing condition of cryptococcosis (CD4^+^ T cell deficiency). Overall, our data support that SGs act as immunoadjuvants to GXM in promoting an early host-protective immune response mediated by γδ T cells via the production of IFNγ and IL-17A in a TLR2-dependent mechanism.

**Fig. 8:**
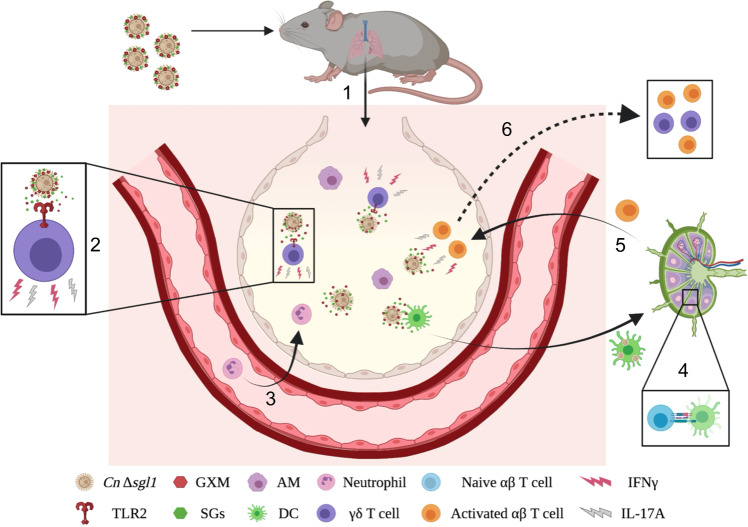
Graphical model illustrating the immune mechanism of host vaccination by *C. neoformans* (*Cn*) Δ*sgl1*. Upon intranasal administration of *C. neoformans* Δ*sgl1*, the yeast cells travel down the bronchioles into the alveoli (1) encountering resident alveolar macrophages (AM), dendritic cells (DCs), and γδ T cells. Both sterylglucosides (SGs) and the glucuronoxylomannan (GXM)-rich capsule of *C. neoformans* Δ*sgl1* can be found on the surface of yeast cells or/and in the extracellular environment. γδ T cells recognize and respond directly to *C. neoformans* Δ*sgl1* via a toll-like receptor 2 (TLR2)-directed mechanism (2) producing the host-protective cytokines IFNγ and IL-17A leading to a pro-inflammatory lung cytokine environment with augmented leukocyte recruitment to the lungs (3) including neutrophils and monocytes that mediate early host control by limiting *C. neoformans* Δ*sgl1* replication. Antigen-presenting cells (APCs) become activated, phagocytose the yeast, and travel to the lung-draining lymph node to prime naive CD4^+^ or CD8^+^ T cells when CD4^+^ T cells are absent (4). The naive T cells differentiate into protectively polarized IFNγ- and IL-17A-producing T cells and travel to the lungs (5) resulting in the pulmonary clearance of the mutant. While most recruited leukocytes die upon resolution of the inflammatory response, a small percentage of αβ and γδ T cells become host-protective memory T cells (6) that rapidly respond upon a subsequent WT challenge, promptly producing both IFNγ and IL-17A and conferring strong vaccine protection. Graphical illustration created with BioRender.com.

A proposed mechanistic model for *C. neoformans* Δ*sgl1*-induced vaccine protection in the host is illustrated in Fig. 8. Upon inhalation of *C. neoformans* Δ*sgl1* into the alveolar spaces of the lungs, γδ T cells quickly recognize and respond to the SGs and GXM from *C. neoformans* Δ*sgl1* with robust production of IFNγ and IL-17A through a TLR2-dependent mechanism sparking an early pro-inflammatory response in the lungs and effector leukocyte recruitment including neutrophils. These γδ T cells and recruited effector cells temporarily control the proliferation and lung containment of *C. neoformans* Δ*sgl1* until the protectively polarized IFNγ- and IL-17A-producing CD4^+^ and/or CD8^+^ T cells migrate back to the lungs solidifying full host control of the mutant. Following host control of the mutant, there is a decrease of inflammatory cytokines and effector cells in the lungs, but a small percentage of αβ and γδ T cells remain as IFNγ- and IL-17A-producing memory T cells. These memory T cells rapidly respond to the WT challenge via the production of IFNγ and IL-17A arming recruited neutrophils for complete host control of the WT strain in immunocompetent, CD4-deficient, or CD8-deficient mice.

Only a limited number of studies have previously focused on the immunogenic properties of SGs, and all of the work was conducted using plant-derived SGs. Nonetheless, these compounds have been shown to stimulate Th1 CD4^+^ T cell proliferation^33^, prolong the survival of mice in a model of disseminated candidiasis^38,51^, work as adjuvants by improving the efficacy of anti-tuberculosis treatments in human patient clinical trials^40^, and display anti-cancer activity by inhibiting tumor invasion and increasing drug effectiveness^52^. Interestingly, we observed an improved protective lymphocyte response defined by type 1 (IFNγ-producing) and type 17 (IL-17A-producing) polarized CD4^+^ and/or CD8^+^ T cells as well as noncanonical γδ T cells upon administration of *C. neoformans* Δ*sgl1*, which accumulates fungal-derived SGs, compared to these previous studies using plant-derived SGs.

While the protective nature of SGs in the work by Lee et al.^38^ and Lee and Han^51^ was also dependent on IFNγ, since all mice depleted of IFNγ prior to SGs administration fully succumbed to infection similar to what we observed here, both immunocompetent and CD4-deficient mice exhibited only ~60–70% survival in these studies while we show complete (100%) survival in both of these conditions. This difference may possibly be attributed to the administration route of the plant-derived SGs in the work by Lee et al.^38^ and Lee and Han^51^. Both administered the SGs intraperitoneally, bypassing any mucosal barrier tissue where γδ T cells would be found and optimally function. Future investigation into the protective nature of fungal-derived SGs alongside another pulmonary invasive fungal pathogen, such as *Aspergillus fumigatus*, is warranted to explore the pan-fungal efficacy of fungal-derived SGs in antifungal vaccine formulations. Overall, these data suggest that the fungal-derived SGs during administration of *C. neoformans* Δ*sgl1* provided an enhanced cellular response and increased host survival than the plant-derived SGs in the context of antifungal protection.

We hypothesized that SGs work as an immunoadjuvant to GXM to produce the observed protective host immune response. It is noteworthy to mention that Lee et al.^38^ and Lee and Han^51^ also solely administered the plant-derived SGs prior to infection and not concurrently with the yeast. If SGs are working as an immunoadjuvant through a T cell-directed mechanism as we hypothesize, the adjuvant should be administered alongside the infection to allow for the generation of optimal host immunity to the immunogenic components of the pathogenic yeast. Nevertheless, we have shown here that the combination of SGs and GXM (*C. neoformans* Δ*sgl1*) produced significant and robust cytokine responses by γδ T cells compared to either GXM alone (*C. neoformans* WT) or SGs alone (*C. neoformans* Δ*cap59*Δ*sgl1*) confirming prior findings that both SGs and GXM are required for host protection^36^.

The capsule of *C. neoformans* is made up of GXM, galactoxylomannan, and mannoproteins^53,54^, although GXM comprises ~90% of the capsule by mass. GXM is composed of an α(1,3) mannose backbone with β(1,2) and β(1,4) xylose and β(1,2) glucuronic acid substitutions^53,55^ that vary depending on the serotype of *Cryptococcus* and hence antigenic differences^56–58^. These serotypes include A, B, C, D, and AD, which differentially group *C. neoformans* (serotypes A, D, and AD) and *C. gattii* (serotypes B and C), and the various *Cryptococcus* serotypes exhibit differing degrees of virulence. Intriguingly, *C. neoformans* Δ*sgl1* has been shown to exhibit serotype-independent protection since *C. neoformans* Δ*sgl1*-vaccinated mice have been shown to be protected from both the highly-virulent WT *C. neoformans* strain H99 serotype A and *C. gattii* strain R265 serotype B^32^. However, investigating the efficacy of SGs as an immunoadjuvant with each individual component of GXM for *C. neoformans* Δ*sgl1* to induce host protection is warranted and currently underway in our lab. This work will allow for the potential production of a more clinically relevant subunit-based vaccine strategy using synthetic formulations of only the necessary fungal components in the future.

Type 1 polarized CD4^+^ T cells have been well-documented to be essential in orchestrating host protection against WT *C. neoformans*^59–63^. Given that HIV/AIDS patients are at high risk for cryptococcosis due to CD4^+^ T cell lymphopenia^3,64^, the role of type 1 CD4^+^ polarized T cells in mediating host protection is undeniable. Although we have reported that either T cell subset sufficiently provided complete protection with no extrapulmonary dissemination after *C. neoformans* Δ*sgl1* vaccination^41^, we have now discovered that CD4^+^ T cells exhibit a more robust response compared to CD8^+^ T cells (Fig. 1). A greater number of type 1 and type 17 polarized CD4^+^ compared to CD8^+^ T cells was observed during immunocompetency (Fig. 1a–d) and during T cell immunodeficiency as well (Fig. 1e–h). However, the IL-17A-producing T cell responses observed in immunocompetent or CD8-deficient mice was attenuated in CD4-deficient mice, which was the only condition where the *C. neoformans* Δ*sgl1*-vaccinated mice had a significantly greater number of IL-13-producing T cells compared to unvaccinated mice. It has been shown using human T cells that CD8^+^ T cell-mediated killing of *C. neoformans* required granulysin that was partially dependent on the presence of CD4^+^ T cells^65^. Although mice do not express granulysin, there may be some correlation between the intrinsic need for CD4^+^ T cells for enhanced CD8^+^ T cell functionality. This may explain the lack of pulmonary clearance of *C. neoformans* Δ*sgl1* in CD4-depleted mice and the significantly higher endpoint lung fungal burden post WT challenge from previous work in our lab^41^.

A type 1 immune response defined by IFNγ production, STAT1 signaling, and classically activated macrophages has been well-documented as an essential component of host protection against *C. neoformans*^66–71^. Although IL-17 has been implicated as a potent antifungal effector cytokine in other fungal infections^72–76^, the requirement for IL-17 in host defense against *C. neoformans* remains uncertain^77–80^. Here, we report that both IFNγ and IL-17A were required for *C. neoformans* Δ*sgl1*-mediated host vaccine protection to the WT strain, regardless of T cell immunodeficiency (Fig. 2) since these essential cytokines were shown to be independently produced by both CD4^+^ and CD8^+^ T cells. Although there was a greater number of IL-17A-producing CD4^+^ and CD8^+^ T cells compared to IFNγ-producing T cells, IFNγ was more determinant of host mortality. All mice neutralized of IFNγ fatally succumbed to WT infection within 20 days, while a ~35% survival rate was observed for mice with abrogated IL-17A signaling either through direct cytokine neutralization (Fig. 2) or IL-17-directed effector cell depletion (i.e., neutrophils (Supplementary Fig. 4D)). Nevertheless, surviving mice all suffered from extrapulmonary dissemination of the yeast to the brain (Supplementary Fig. 4B, C) indicating these mice lacked pulmonary containment and may have succumbed to illness over time.

The loss of pulmonary containment in the absence of IL-17A may be explained in part from the coordinated effects of IL-22 alongside of IL-17. IL-22 is a cytokine produced by several innate and adaptive immune cells including IL-17A-producing CD4^+^ and CD8^+^ T cells, γδ T cells, and type 3 innate lymphoid cells, among others^81–84^, which functions through promoting epithelial tissue integrity at mucosal barrier tissues. Since we have observed here and in our prior work^41^ that *C. neoformans* Δ*sgl1*-vaccinated mice are fully protected yet exhibit a consistent number of persistent WT cells in the lungs post challenge that do not disseminate, increased epithelial tissue integrity via IL-22 may be a rationale for this containment. In addition, it has also been shown that IL-22 production by innate lymphoid cells in the lungs prevented experimental neutrophilic inflammation^85^, so the resolution of inflammation and inflammatory cell recruitment observed in *C. neoformans* Δ*sgl1*-vaccinated mice may also have some attribution to IL-22 signaling although future investigation into this cytokine in our vaccination model is required.

In addition to the aforementioned canonical T cells, γδ T cells are innate-like lymphocytes that are potent sources of IL-17A and/or IFNγ at mucosal barrier tissues that have been studied mainly in the context of bacterial infections^46,48,86,87^ or cancer^88–90^, yet the role of γδ T cells during cryptococcal infections remains largely understudied^45,91^. Uezu and colleagues observed a significant decrease in the lung fungal burden and a significant increase in lung and serum levels of IFNγ in TCRδ^−/−^ mice during infection with *C. neoformans*^45^. Similarly, Wozniak and colleagues reported that IL-17A production by γδ T cells was only essential when neutrophils were depleted during infection with *C. neoformans*^91^. In the present study however, γδ T cells are considered to be the cornerstone of early lung recognition supported by the fact that *C. neoformans* Δ*sgl1* was neither cleared (Fig. 3d) nor contained to the lungs (Supplementary Fig. 6) in TCRδ^−/−^ mice even though CD4^+^ T cells and all required effector cytokines were still present. Remarkably, we have shown here that ex vivo cultured splenocytes from TCRβ^−/−^ mice (mainly consisting of γδ T cells) or purified γδ T cells produced significantly greater amounts of IFNγ and IL-17A when stimulated with live or HK *C. neoformans* Δ*sgl1* compared to either live or HK WT strain or *C. neoformans* Δ*cap59*Δ*sgl1* (Fig. 4a–d) with or without plate-bound anti-TCRγδ monoclonal antibody (Fig. 4e, f and Supplementary Fig. 7). This robust production of IFNγ and IL-17A by γδ T cells suggests that the early innate response to our mutant in the lungs was strongly dependent on both SGs and GXM in support of our hypothesis that SGs act as an immunoadjuvant to GXM for induction of protective host immunity.

γδ T cells have also been reported to be able to form memory populations post antigen encounter^87,92,93^. Quantification of γδ T cells post WT challenge in *C. neoformans* Δ*sgl1*-vaccinated and unvaccinated mice showed that there was a significantly greater number of γδ T cells in vaccinated mice on day 7 post WT challenge during immunocompetency (Fig. 3a), CD8-deficiency (Fig. 3b), and CD4-deficiency (Fig. 3c) compared to unvaccinated mice, which was also observed during intracellular cytokine production of T cells on days 3 and 7 post challenge (Fig. 6d–f). We then found that γδ T cells were required for host protection upon WT challenge (Fig. 3e) highlighting their necessity in *C. neoformans* Δ*sgl1*-vaccinated mice. Therefore, γδ T cells are now implicated to be essential in sparking an early pro-inflammatory immune response in the lungs upon administration of *C. neoformans* Δ*sgl1* (Fig. 6a–c) and for host protection upon WT challenge (Fig. 6d–f).

Studies have shown that γδ T cells are required to direct neutrophil recruitment and their subsequent effector functions via IL-17 signaling during bacterial infections^94,95^. Since we have now shown here that neutrophils are required for host protection against the WT strain in *C. neoformans* Δ*sgl1*-vaccinated mice (Supplementary Fig. 4D), IL-17A from γδ T cells may be the driving factor for neutrophil recruitment and functioning in our model as well. To better understand the long-term memory potential of γδ T cells and neutrophil-directed means of protection, phenotypic characterization of γδ T cells post-vaccination will be explored in future studies.

Since γδ T cells can form long-lived memory populations, the potential for these cells as immunotherapeutic agents has been highly considered. In the case of cryptococcosis, HIV^+^/AIDS patients are the most susceptible population, and γδ T cells have been shown to remain functional in reported cases of human of CD4-lymphopenia^90,96^. Intriguingly, one study reported that γδ T cells taken from HIV-1-infected patients were shown to produce similar levels of both IFNγ and IL-17 when cultured ex vivo compared to γδ T cells taken from healthy donors in response to *C. albicans*^43^. Thus, γδ T cell-mediated vaccination with *C. neoformans* Δ*sgl1* may be able to provide efficacious immunotherapeutic potential for HIV^+^/AIDS patients in the future.

TLR2 is correlated with activation of the NF-κB pathway resulting in pro-inflammatory cytokine production including TNFα, IL-12, IFNγ, IL-18, IL-1β, and IL-17A^97^, and has been implicated in the protective host immune response to *C. neoformans* WT infection^98–100^ as well as for the effector functions of γδ T cells^46,47,50,101^. Capsular GXM from *C. neoformans* has been shown to interact with host cell PRRs, including TLR2, but in an immunosuppressive manner, by dampening the pro-inflammatory cytokine expression or induction of the Fas ligand on phagocytes^102–104^. Here, we found that host recognition and response to the SGs and GXM from *C. neoformans* Δ*sgl1* was dependent upon TLR2, and the presence of GXM was necessary to stimulate cytokine production (Figs. 5 and 6). In addition, ex vivo cultured γδ T cells from TLR2^−/−^ mice exhibited an attenuated IFNγ response and a nearly absent IL-17A response compared to γδ T cells from C57BL/6 mice upon stimulation with live or HK *C. neoformans* Δ*sgl1*, indicating that TLR2 was required for host recognition and response to *C. neoformans* Δ*sgl1*. Interestingly, the IL-17A response was not statistically different in γδ T cells from TLR2^−/−^ mice stimulated with *C. neoformans* Δ*sgl1* compared to γδ T cells from TLR2^−/−^ mice stimulated with the WT strain or *C. neoformans* Δ*cap59*Δ*sgl1* (Fig. 5), which strongly supports the hypothesis that SGs act as an immunoadjuvant to GXM in positively modulating TLR2 for a protective host immune response.

The protective response induced by the TLR2-dependent mechanism was shown to be required for host protection as well. Foremost, we have shown that γδ T cells fail to produce IFNγ and IL-17A when lacking TLR2 both ex vivo (Fig. 5) as well as in vivo (Fig. 6). In terms of in vivo survivability, all vaccinated TLR2^−/−^ mice fatally succumbed to WT infection post challenge at a nearly identical rate to unvaccinated TLR2^−/−^ mice (Fig. 7). Host signaling through surface receptors, including TLRs, has been suggested to require functional lipid raft microdomains on the plasma membrane of host cells that concentrate receptors to a common region for increased signaling capabilities^105–109^. These lipid rafts are mediated via host sphingolipids found in the plasma membrane, most notably including sphingomyelin. In fact, a sphingomyelin deficiency, such as with sphingomyelin synthase knockout mice or pharmacological depletion of sphingomyelin from the plasma membrane, has been shown to impair T cell receptor signaling^110^, inhibit phagocytosis of *C. neoformans*^105^, decrease the antimicrobial activity of neutrophils against *C. neoformans*^111^, and attenuate NF-κB activation^112^. Thus, lipid raft formation for host cell signaling represents an important host factor for vaccination with *C. neoformans* Δ*sgl1*. The role of sphingomyelin synthase activity with regards to TLR2 signaling and downstream cytokine production is currently under investigation in our lab. Taken together, TLR2 is an essential host factor for both recognition and response to *C. neoformans* Δ*sgl1* as well as host protection from subsequent WT challenge in vaccinated mice.

To our knowledge, this is the first report that examines fungal-derived SGs as immunoadjuvants stimulating host-protective γδ T cells that produce IFNγ and IL-17A through a TLR2-dependent mechanism. Given that *C. neoformans* Δ*sgl1* is avirulent and confers complete host protection in numerous models of clinically relevant immunodeficiency, our novel data highlight the host immune mechanisms required to protect at-risk individuals from invasive fungal infections, such as cryptococcosis, potentially leading to improved antifungal vaccine research and development.

## Materials and methods

### Fungal strains and heat-killed (HK) yeast preparation

The fungal strains used in this study included the wild-type *C. neoformans* var. *grubii* strain H99 serotype A (WT), *C. neoformans* Δ*sgl1*, a mutant strain accumulating sterylglucosides (SGs)^32^, and *C. neoformans* Δ*cap59*Δ*sgl1*, an acapsular mutant accumulating SGs^36^. For all experiments, fungal strains were recovered from a −80 °C freezer stock on YPD plates at 30 °C for 3–4 days. An isolated colony was added to 10 ml of YPD broth and grown at 30 °C for 16–18 h with shaking, washed with sterile PBS, counted on a hemocytometer, and resuspended in sterile PBS at the desired concentration. For HK strains, the desired concentration of live yeast was resuspended in PBS and added to an 80 °C heat block for 75 min. All HK inoculums were confirmed to be killed by plating the mixture on YPD plates at 30 °C for 3–4 days and observing no growth.

### Mice and ethical statement

Male and female CBA/JCrHsd mice (stock #5501) were purchased from Envigo. Male and female C57BL/6 (strain #000664), female TCRβ^−/−^ (B6.129P2-*Tcrb*^*tm1Mom*^/J; strain #002118), female TCRδ^−/−^ (B6.129P2-*Tcrd*^*tm1Mom*^/J; strain #002120), and female TLR2^−/−^ (B6.129-*Tlr2*^*tm1Kir*^/J; strain #004650) mice were purchased from The Jackson Laboratory. All animals were housed 3–4 animals per cage under specific pathogen-free conditions and had access to food and water *ad libitum*. Mice were allowed one week to acclimate upon arrival before any procedures began. All animal procedures were approved by the Stony Brook University Institutional Animal Care and Use Committee (protocol no. 341588) and followed the guidelines of the American Veterinary Medical Association.

### In vivo monoclonal antibody depletions

All antibodies for depletion of specific cell populations or neutralization of specific cytokines were purchased from BioXCell. Administration of depletion antibodies was performed 48 h prior to infection and continued the length of the experiment at set intervals to maintain the desired effect as previously determined^41^. All depletion antibodies were isotype-matched for administration to control groups. The specific time intervals, concentrations, clones, and target populations for each antibody can be found in Table 1.

**Table 1:** Antibodies used for in vivo cell depletions and cytokine neutralizations.

Antibody	Clone	Concentration	Administration	Target
Anti-Ly6G	1A8	225 μg/100 μl	Every 2d IP	Neutrophils
Anti-CD8	116-13.1	400 μg/100 μl	Every 4d IP	CD8^+^ T cells
Anti-CD4	GK1.5	400 μg/100 μl	Every 4d IP	CD4^+^ T cells
Anti-IL-17A	17F3	250 μg/100 μl	Every 4d IP	Exogenous IL-17A
Anti-IFNγ	XMG1.2	300 μg/100 μl	Every 4d IP	Exogenous IFNγ

*IP* intraperitoneally.

### Infection, survival studies, and organ fungal burden

Mice were first intraperitoneally (IP) anesthetized with a ketamine/xylazine solution (95 mg of ketamine and 5 mg of xylazine per kg of animal body weight). Anesthetized mice were intranasally (IN) immunized with 5 × 10^5^ CFU *C. neoformans* Δ*sgl1* in 20 μl of PBS or 20 μl of sterile PBS (unvaccinated controls), challenged 30 days later with 5 × 10^5^ CFU *C. neoformans* WT in 20 μl of PBS, and monitored daily until the pre-determined experimental endpoint where mice were euthanized via CO_2_. Any animal that appeared to be moribund, exhibited labored breathing or neurological infection, or had lost more than 20% body weight was euthanized via CO_2_. For fungal burden analysis, mice were euthanized via CO_2_ at specified timepoints. The lungs, brain, spleen, kidneys, and liver were removed, homogenized in 10 ml of sterile PBS using a Stomacher 80 blender (Seward, UK), and serial dilutions were grown on YPD plates at 30 °C for 2–3 days prior to counting and calculation of total organ fungal burden.

### Flow cytometry

Flow cytometry was used to quantify lung leukocyte populations. Briefly, lungs were removed from mice euthanized under CO_2_, minced, and incubated at 37 °C with 5% CO_2_ for 90 min with shaking in FACS buffer supplemented with collagenase IV and DNase I. The digested tissue was homogenized through a 70 μm pore filter, RBCs lysed with Ack lysis buffer, washed, and filtered through a 70 μm pore filter into single-cell suspensions in FACS buffer. Cells were counted and 10^6^ live cells were Fc blocked for 20 min in the dark on ice, then surface stained using a cocktail of direct fluorochrome-conjugated antibodies for 30 min in the dark on ice. For intracellular cytokine staining of T cells, 10^6^ live lung cells were resuspended in stimulation media [RPMI with 10% fetal bovine serum (FBS), 1% penicillin and streptomycin, 0.1 μg/ml anti-CD3, 1 μg/ml anti-CD28, and 1 μl/ml Brefeldin A (BioLegend)] or [RPMI with 10% FBS, 1% penicillin and streptomycin, and 2 μl/ml of cell activation cocktail with Brefeldin A (BD Biosciences)], incubated for 5–6 h at 37 °C with 5% CO_2_ to induce cytokine production, washed, Fc blocked, surface stained, fixed and permeabilized using BD Cytofix/Cytoperm Plus (BD Biosciences), stained for intracellular cytokines with an antibody cocktail for 1 h in the dark on ice, and run on an LSR II flow cytometer (BD Biosciences). All data were analyzed using Flowjo v10. The antibody-fluorochrome combinations (all purchased from BioLegend) included: viability-AF700; CD45-BV711; CD4-BV785; CD8-BV605; TCRγδ-BV510; CD3-APC cy7; IFNγ-FITC; IL-17A-PE; IL-13-APC cy7. The gating scheme for each individual population is shown in the Supplementary data.

### Ex vivo splenocyte stimulation and ELISA

96-well plates were incubated with or without 100 µl of diluted anti-TCRγδ antibody (BioXCell; clone: UC7-13D5; concentration: 4 µg/ml) at 37 °C and 5% CO_2_ for 4 h to bind the antibody to the plate and washed twice with PBS to remove unbound antibody. Spleens were harvested from either uninfected C57BL/6, TCRβ^−/−^, or TLR2^−/−^ mice, lysed of RBCs, washed, and processed for single-cell suspensions through a 70 μm pore filter. These cells were counted and either (i) 10^5^ live splenocytes in 100 µl complete RPMI were seeded into a 96-well plate with plate-bound anti-TCRγδ antibody or (ii) 10^5^ live γδ T cells were purified in 100 µl complete RPMI via a mouse γδ T cell isolation MACS separation kit (Miltenyi Biotec; catalog number 130-092-125) using an LD and MS column according to the manufacturer’s directions. 10^5^ live or HK *C. neoformans* WT, *C. neoformans* Δ*sgl1*, *C. neoformans* Δ*cap59*Δ*sgl1*, or sterile PBS in 100 µl of complete RPMI were added to the appropriate wells in a 1:1 ratio to the γδ T cells. Seeded plates were incubated at 37 °C and 5% CO_2_ for 5 days. Supernatants were collected 1, 3, and 5 days post incubation and stored at −80 °C until the ELISA was performed. The supernatants were divided and examined for IFNγ and IL-17A production using Legend Max Mouse IFNγ or IL-17A ELISA kits (BioLegend) following the manufacturer’s instructions exactly.

### Statistical analysis and study design

All statistical analyses were performed using GraphPad Prism 9 software. The sample size, statistical analysis, and statistical significance are all described for each figure in the figure captions. No data was excluded from statistical analysis. One to three experimental replicates were carried out for all ex vivo datasets, and a representative figure was shown for each experiment. For in vivo survival studies, ten mice per group were used for statistical power and to reduce the number of mice used throughout the study. The Mantel–Cox log-rank test was used to calculate significance for survival studies. A two-way ANOVA using Šídák’s multiple comparisons test for *P* value adjustment was used to calculate statistical significance between more than two samples and represented as the mean ± standard deviation (SD).

## Supplementary information

Supplementary information

## References

[1] Pathakumari B, Liang G, Liu W (2020). Immune defence to invasive fungal infections: a comprehensive review. Biomed. Pharmacother..

[2] Fierer J (2019). Invasive endemic fungi of the Western Hemisphere. Virulence.

[3] Akhtar S, Aggarwal N, Demkowicz R, Andreatos N, Gupta M (2020). Cryptococcus and HIV. QJM.

[4] Zhao Y, Lin X (2021). *Cryptococcus neoformans*: sex, morphogenesis, and virulence. Infect. Genet Evol..

[5] Mayer FL, Kronstad JW (2020). *Cryptococcus neoformans*. Trends Microbiol..

[6] Maziarz EK, Perfect JR (2016). Cryptococcosis. Infect. Dis. Clin. N. Am..

[7] Henao-Martinez AF, Beckham JD (2015). Cryptococcosis in solid organ transplant recipients. Curr. Opin. Infect. Dis..

[8] Saha DC (2007). Serologic evidence for reactivation of cryptococcosis in solid-organ transplant recipients. Clin. Vaccin. Immunol..

[9] Bryan AM (2020). FTY720 reactivates cryptococcal granulomas in mice through S1P receptor 3 on macrophages. J. Clin. Investig..

[10] Grebenciucova E, Reder AT, Bernard JT (2016). Immunologic mechanisms of fingolimod and the role of immunosenescence in the risk of cryptococcal infection: a case report and review of literature. Mult. Scler. Relat. Disord..

[11] Ward MD, Jones DE, Goldman MD (2016). Cryptococcal meningitis after fingolimod discontinuation in a patient with multiple sclerosis. Mult. Scler. Relat. Disord..

[12] Del Poeta M (2022). Cryptococcal meningitis reported with Fingolimod treatment: case series. Neurol. Neuroimmunol. Neuroinflammation..

[13] Cogliati M (2021). Global warming impact on the expansion of fundamental niche of *Cryptococcus gattii* VGI in Europe. Environ. Microbiol Rep..

[14] de SAraujo GR, Souza W, Frases S (2017). The hidden pathogenic potential of environmental fungi. Future Microbiol..

[15] Raffa RB, Eltoukhy NS, Raffa KF (2012). Implications of climate change (global warming) for the healthcare system. J. Clin. Pharm. Ther..

[16] van Rhijn N, Bromley M (2021). The consequences of our changing environment on life threatening and debilitating fungal diseases in humans. J. Fungi.

[17] Brunet K, Alanio A, Lortholary O, Rammaert B (2018). Reactivation of dormant/latent fungal infection. J. Infect..

[18] Shibuya K (2005). Granuloma and cryptococcosis. J. Infect. Chemother..

[19] Zhao Y, Lin J, Fan Y, Lin X (2019). Life cycle of *Cryptococcus neoformans*. Annu. Rev. Microbiol..

[20] Diaz JH (2020). The disease ecology, epidemiology, clinical manifestations, and management of emerging *Cryptococcus gattii* complex infections. Wilderness Environ. Med..

[21] Chang CC, Chen SC (2015). Colliding epidemics and the rise of Cryptococcosis. J. Fungi.

[22] Montoya MC, Magwene PM, Perfect JR (2021). Associations between Cryptococcus genotypes, phenotypes, and clinical parameters of human disease: a review. J. Fungi.

[23] Rajasingham R (2017). Global burden of disease of HIV-associated cryptococcal meningitis: an updated analysis. Lancet Infect. Dis..

[24] Bicanic T (2015). Toxicity of amphotericin B deoxycholate-based induction therapy in patients with HIV-associated cryptococcal meningitis. Antimicrob. Agents Chemother..

[25] McEvoy K, Normile TG, Poeta MD (2020). Antifungal drug development: targeting the fungal sphingolipid pathway. J. Fungi.

[26] Nami S (2019). Fungal vaccines, mechanism of actions and immunology: a comprehensive review. Biomed. Pharmacother..

[27] Mourad A, Perfect JR (2018). Present and future therapy of cryptococcus infections. J. Fungi.

[28] Ueno K, Yanagihara N, Shimizu K, Miyazaki Y (2020). Vaccines and protective immune memory against Cryptococcosis. Biol. Pharm. Bull..

[29] Caballero Van Dyke MC, Wormley FL (2018). A call to arms: quest for a cryptococcal vaccine. Trends Microbiol..

[30] Gushiken AC, Saharia KK, Baddley JW (2021). Cryptococcosis. Infect. Dis. Clin. N. Am..

[31] Normile TG, Bryan AM, Del, Poeta M (2020). Animal models of *Cryptococcus neoformans* in Identifying immune parameters associated with primary infection and reactivation of latent infection. Front Immunol..

[32] Rella A (2015). Role of Sterylglucosidase 1 (Sgl1) on the pathogenicity of *Cryptococcus neoformans*: potential applications for vaccine development. Front. Microbiol..

[33] Bouic P (1996). Beta-sitosterol and beta-sitosterolglucoside stimulate human peripheral blood lymphocyte proliferation: Implications for their use as an immunomodulatory vitamin combination. Int J. Immunopharmac..

[34] Grille S, Zaslawski A, Thiele S, Plat J, Warnecke D (2010). The functions of steryl glycosides come to those who wait: recent advances in plants, fungi, bacteria and animals. Prog. Lipid Res..

[35] Normile TG, McEvoy K, Del Poeta M (2020). Steryl glycosides in fungal pathogenesis: an understudied immunomodulatory adjuvant. J. Fungi.

[36] Colombo AC (2019). Cryptococcus neoformans glucuronoxylomannan and sterylglucoside are required for host protection in an animal vaccination model. mBio.

[37] Pereira de Sa N (2021). Structure and inhibition of *Cryptococcus neoformans* sterylglucosidase to develop antifungal agents. Nat. Commun..

[38] Lee JH (2007). Immunoregulatory activity by daucosterol, a beta-sitosterol glycoside, induces protective Th1 immune response against disseminated Candidiasis in mice. Vaccine.

[39] Kasirzadeh S (2021). beta-Sitosterol alters the inflammatory response in CLP rat model of sepsis by modulation of NFkappaB signaling. Biomed. Res. Int..

[40] Donald P (1997). A randomised placebo-controlled trial of the efficacy of beta-sitosterol and its glucoside as adjuvants in the treatment of pulmonary tuberculosis. Int. J. Tuberculosis Lung Dis..

[41] Normile TG, Rella A, Del Poeta M (2021). Cryptococcus neoformans Delta-sgl1 vaccination requires either CD4+ or CD8+ T cells for complete host protection. Front. Cell. Infect. Microbiol..

[42] Normile TG, Del Poeta M (2022). Three models of vaccination strategies against Cryptococcosis in immunocompromised hosts using heat-killed *Cryptococcus neoformans* Δsgl1. Front. Immunol.

[43] Fenoglio D (2009). Vdelta1 T lymphocytes producing IFN-gamma and IL-17 are expanded in HIV-1-infected patients and respond to *Candida albicans*. Blood.

[44] Martin B, Hirota K, Cua DJ, Stockinger B, Veldhoen M (2009). Interleukin-17-producing gammadelta T cells selectively expand in response to pathogen products and environmental signals. Immunity.

[45] Uezu K (2004). Accumulation of gammadelta T cells in the lungs and their regulatory roles in Th1 response and host defense against pulmonary infection with *Cryptococcus neoformans*. J. Immunol..

[46] Mokuno Y (2000). Expression of toll-like receptor 2 on gamma delta T cells bearing invariant V gamma 6/V delta 1 induced by *Escherichia coli* infection in mice. J. Immunol..

[47] Dar AA, Patil RS, Chiplunkar SV (2014). Insights into the relationship between toll like receptors and gamma delta T cell responses. Front. Immunol..

[48] Moore TA, Moore BB, Newstead MW, Standiford TJ (2000). Gamma delta-T cells are critical for survival and early proinflammatory cytokine gene expression during murine *Klebsiella pneumonia*. J. Immunol..

[49] McKenzie DR (2017). IL-17-producing gammadelta T cells switch migratory patterns between resting and activated states. Nat. Commun..

[50] Wesch D, Peters C, Oberg HH, Pietschmann K, Kabelitz D (2011). Modulation of gammadelta T cell responses by TLR ligands. Cell Mol. Life Sci..

[51] Lee JH, Han Y (2006). Ginsenoside Rg1 helps mice resist to disseminated candidiasis by Th1 type differentiation of CD4+ T cell. Int. Immunopharmacol..

[52] Chou FP (2017). An enzymatic approach to configurationally rare trans-androsteronyl-alpha-glucoside and Its potential anticancer application. Chem. Biol. Drug Des..

[53] Agustinho DP, Miller LC, Li LX, Doering TL (2018). Peeling the onion: the outer layers of *Cryptococcus neoformans*. Mem. Inst. Oswaldo Cruz..

[54] Teixeira PA, Penha LL, Mendonca-Previato L, Previato JO (2014). Mannoprotein MP84 mediates the adhesion of *Cryptococcus neoformans* to epithelial lung cells. Front. Cell. Infect. Microbiol..

[55] Kuttel MM, Casadevall A, Oscarson S (2020). *Cryptococcus neoformans* capsular GXM conformation and epitope presentation: a molecular modelling study. Molecules.

[56] Probert M (2019). A glucuronoxylomannan epitope exhibits serotype-specific accessibility and redistributes towards the capsule surface during titanization of the fungal pathogen *Cryptococcus neoformans*. Infect. Immun..

[57] Wang ZA, Li LX, Doering TL (2018). Unraveling synthesis of the cryptococcal cell wall and capsule. Glycobiology.

[58] Zaragoza O (2009). The capsule of the fungal pathogen *Cryptococcus neoformans*. Adv. Appl Microbiol..

[59] Neal LM (2017). T cell-restricted notch signaling contributes to pulmonary Th1 and Th2 immunity during *Cryptococcus neoformans* Infection. J. Immunol..

[60] Wozniak KL, Young ML, Wormley FL (2011). Protective immunity against experimental pulmonary cryptococcosis in T cell-depleted mice. Clin. Vaccin. Immunol..

[61] Chaturvedi AK, Wormley FL (2017). Methodology for anti-cryptococcal vaccine development. Methods Mol. Biol..

[62] Lindell DM, Moore TA, McDonald RA, Toews GB, Huffnagle GB (2005). Generation of antifungal effector CD8+ T cells in the absence of CD4+ T cells during *Cryptococcus neoformans* infection. J. Immunol..

[63] Mukaremera L, Nielsen K (2017). Adaptive immunity to *Cryptococcus neoformans* infections. J. Fungi.

[64] Linyu L, Ali Abuderman AW, Muzaheed, Acharya S, Divakar DD (2020). Modulation of host immune status by cryptococcus co-infection during HIV-1 pathogenesis and its impact on CD+4 cell and cytokines environment. Micro. Pathog..

[65] Ma LL (2002). CD8 T cell-mediated killing of *Cryptococcus neoformans* requires granulysin and is dependent on CD4 T cells and IL-15. J. Immunol..

[66] Leopold Wager CM (2018). IFN-gamma immune priming of macrophages in vivo induces prolonged STAT1 binding and protection against *Cryptococcus neoformans*. PLoS Pathog..

[67] Leopold Wager CM (2015). STAT1 signaling within macrophages is required for antifungal activity against *Cryptococcus neoformans*. Infect. Immun..

[68] Leopold Wager CM, Hole CR, Wozniak KL, Olszewski MA, Wormley FL (2014). STAT1 signaling is essential for protection against *Cryptococcus neoformans* infection in mice. J. Immunol..

[69] Wozniak KL, Hardison S, Olszewski M, Wormley FL (2012). Induction of protective immunity against cryptococcosis. Mycopathologia.

[70] Hardison SE (2012). Protective immunity against pulmonary cryptococcosis is associated with STAT1-mediated classical macrophage activation. J. Immunol..

[71] Wormley FL, Perfect JR, Steele C, Cox GM (2007). Protection against cryptococcosis by using a murine gamma interferon-producing *Cryptococcus neoformans* strain. Infect. Immun..

[72] Aggor FEY (2020). Oral epithelial IL-22/STAT3 signaling licenses IL-17-mediated immunity to oral mucosal candidiasis. Sci. Immunol.

[73] Li T, Rong HM, Zhang C, Zhai K, Tong ZH (2018). IL-9 deficiency promotes pulmonary Th17 response in murine model of pneumocystis infection. Front. Immunol..

[74] Nanjappa SG (2017). Antifungal Tc17 cells are durable and stable, persisting as long-lasting vaccine memory without plasticity towards IFNgamma cells. PLoS Pathog..

[75] Nanjappa SG (2015). Intrinsic MyD88-Akt1-mTOR signaling coordinates disparate Tc17 and Tc1 responses during vaccine immunity against fungal pneumonia. PLoS Pathog..

[76] Hernandez-Santos N (2013). Th17 cells confer long-term adaptive immunity to oral mucosal *Candida albicans* infections. Mucosal Immunol..

[77] Wozniak KL, Hardison SE, Kolls JK, Wormley FL (2011). Role of IL-17A on resolution of pulmonary C. neoformans infection. PLoS ONE.

[78] Hardison SE, Wozniak KL, Kolls JK, Wormley FL (2010). Interleukin-17 is not required for classical macrophage activation in a pulmonary mouse model of *Cryptococcus neoformans* infection. Infect. Immun..

[79] Sato K (2020). Production of IL-17A at innate immune phase leads to decreased Th1 immune response and attenuated host defense against infection with *Cryptococcus deneoformans*. J. Immunol..

[80] Murdock BJ, Huffnagle GB, Olszewski MA, Osterholzer JJ (2014). Interleukin-17A enhances host defense against cryptococcal lung infection through effects mediated by leukocyte recruitment, activation, and gamma interferon production. Infect. Immun..

[81] Colonna M (2009). Interleukin-22-producing natural killer cells and lymphoid tissue inducer-like cells in mucosal immunity. Immunity.

[82] Gurczynski SJ, Moore BB (2018). IL-17 in the lung: the good, the bad, and the ugly. Am. J. Physiol. Lung Cell Mol. Physiol..

[83] McAleer JP, Kolls JK (2014). Directing traffic: IL-17 and IL-22 coordinate pulmonary immune defense. Immunol. Rev..

[84] Ronacher K, Sinha R, Cestari M (2018). IL-22: an underestimated player in natural resistance to tuberculosis?. Front Immunol..

[85] Felton JM (2018). Facilitation of IL-22 production from innate lymphoid cells by prostaglandin E2 prevents experimental lung neutrophilic inflammation. Thorax.

[86] Murakami T, Hatano S, Yamada H, Iwakura Y, Yoshikai Y (2016). Two types of interleukin 17A-producing gammadelta T cells in protection against pulmonary infection with *Klebsiella pneumoniae*. J. Infect. Dis..

[87] Romagnoli PA, Sheridan BS, Pham QM, Lefrancois L, Khanna KM (2016). IL-17A-producing resident memory gammadelta T cells orchestrate the innate immune response to secondary oral Listeria monocytogenes infection. Proc. Natl Acad. Sci. USA.

[88] Beck BH (2010). Adoptively transferred ex vivo expanded gammadelta-T cells mediate in vivo antitumor activity in preclinical mouse models of breast cancer. Breast Cancer Res. Treat..

[89] Coffelt SB (2015). IL-17-producing gammadelta T cells and neutrophils conspire to promote breast cancer metastasis. Nature.

[90] Latha TS (2014). gammadelta T cell-mediated immune responses in disease and therapy. Front. Immunol..

[91] Wozniak KL, Kolls JK, Wormley FL (2012). Depletion of neutrophils in a protective model of pulmonary cryptococcosis results in increased IL-17A production by gammadelta T cells. BMC Immunol..

[92] Bonneville M, O’Brien RL, Born WK (2010). Gammadelta T cell effector functions: a blend of innate programming and acquired plasticity. Nat. Rev. Immunol..

[93] Khairallah C, Chu TH, Sheridan BS (2018). Tissue adaptations of memory and tissue-resident gamma delta T cells. Front. Immunol..

[94] Nakasone C (2007). Accumulation of gamma/delta T cells in the lungs and their roles in neutrophil-mediated host defense against pneumococcal infection. Microbes Infect..

[95] Shibata K, Yamada H, Hara H, Kishihara K, Yoshikai Y (2007). Resident Vdelta1+ gammadelta T cells control early infiltration of neutrophils after *Escherichia coli* infection via IL-17 production. J. Immunol..

[96] Juno JA, Eriksson EM (2019). gammadelta T-cell responses during HIV infection and antiretroviral therapy. Clin. Transl. Immunol..

[97] De Nardo D (2015). Toll-like receptors: activation, signalling and transcriptional modulation. Cytokine.

[98] Biondo C (2005). MyD88 and TLR2, but not TLR4, are required for host defense against *Cryptococcus neoformans*. Eur. J. Immunol..

[99] Nakamura K (2006). Limited contribution of Toll-like receptor 2 and 4 to the host response to a fungal infectious pathogen, *Cryptococcus neoformans*. FEMS Immunol. Med. Microbiol..

[100] Yauch LE, Mansour MK, Shoham S, Rottman JB, Levitz SM (2004). Involvement of CD14, toll-like receptors 2 and 4, and MyD88 in the host response to the fungal pathogen *Cryptococcus neoformans* in vivo. Infect. Immun..

[101] Dejima T (2011). Protective role of naturally occurring interleukin-17A-producing gammadelta T cells in the lung at the early stage of systemic candidiasis in mice. Infect. Immun..

[102] Burgel PH (2020). *Cryptococcus neoformans* secretes small molecules that inhibit IL-1beta inflammasome-dependent secretion. Mediators Inflamm..

[103] Decote-Ricardo D (2019). Immunomodulatory role of capsular polysaccharides constituents of *Cryptococcus neoformans*. Front. Med..

[104] Monari C (2005). *Cryptococcus neoformans* capsular glucuronoxylomannan induces expression of fas ligand in macrophages. J. Immunol..

[105] Bryan AM (2021). Cholesterol and sphingomyelin are critical for Fcgamma receptor-mediated phagocytosis of *Cryptococcus neoformans* by macrophages. J. Biol. Chem..

[106] Bryan AM, Del Poeta M, Luberto C (2015). Sphingolipids as regulators of the phagocytic response to fungal infections. Mediators Inflamm..

[107] Bryan AM, Farnoud AM, Mor V, Del Poeta M (2014). Macrophage cholesterol depletion and its effect on the phagocytosis of *Cryptococcus neoformans*. J. Vis. Exp..

[108] Prymas K (2020). Sphingomyelin synthase activity affects TRIF-dependent signaling of Toll-like receptor 4 in cells stimulated with lipopolysaccharide. Biochim Biophys. Acta Mol. Cell Biol. Lipids.

[109] Xue J (2019). Sphingomyelin synthase 2 inhibition ameliorates cerebral ischemic reperfusion injury through reducing the recruitment of toll-like receptor 4 to lipid rafts. J. Am. Heart Assoc..

[110] Jin ZX (2008). Impaired TCR signaling through dysfunction of lipid rafts in sphingomyelin synthase 1 (SMS1)-knockdown T cells. Int Immunol..

[111] Qureshi A (2010). Role of sphingomyelin synthase in controlling the antimicrobial activity of neutrophils against *Cryptococcus neoformans*. PLoS ONE.

[112] Hailemariam TK (2008). Sphingomyelin synthase 2 deficiency attenuates NFkappaB activation. Arterioscler Thromb. Vasc. Biol..

