# Supplementary material for: Vaccine protection by Cryptococcus neoformans Δsgl1 is mediated by γδ T cells via TLR2 signaling
Source: Mucosal Immunol. 2022 Oct 13;15(6):1416–30. doi: 10.1038/s41385-022-00570-3 (PMC9705245; doi:10.1038/s41385-022-00570-3)

Supplementary information

Vaccine protection by *Cryptococcus neoformans* Δ*sgl1* is mediated by γδ T cells via TLR2 signaling

Tyler G. Normile,[1] Timothy H. Chu,[1] Brian S. Sheridan,[1] Maurizio Del Poeta[1,2,3]*

[1]Department of Microbiology and Immunology, Stony Brook University, Stony Brook, NY 11794; [2]Division of Infectious Diseases, School of Medicine, Stony Brook University, Stony Brook, NY 11794; [3]Veterans Administration Medical Center, Northport, NY 11768 USA

***Correspondence**: Maurizio Del Poeta, M.D.

Address: 150 Life Science Building, Stony Brook, NY 11794 USA; Fax: (631) 632-9797; Tel: (631) 632-4024; Email: maurizio.delpoeta@stonybrook.edu

Conflict of interest: Dr. Maurizio Del Poeta, M.D., is a Co-Founder and the Chief Scientific Officer (CSO) of MicroRid Technologies Inc.

Author contributions: TGN and MDP took part in the conceptualization of this study and writing of the manuscript. TGN performed all experimental procedures, statistical analyses, and figure generation. TGN, THC, BSS, and MDP partook in experimental planning and finalization of the manuscript.

Acknowledgments: This work was supported by the National Institute of Health (NIH) grants AI136934 (MDP), AI116420 (MDP), and AI125770 (MDP), by a Merit Review Grant I01BX002924 (MDP) from the Veterans Affairs (VA) Program, and by the Department of Defense (DOD) grant PR190642 (MDP). Maurizio Del Poeta is a recipient of the Research Career Scientist (RCS) Award (IK6 BX005386), and a Burroughs Welcome Investigator in Infectious Diseases.

Keywords: *Cryptococcus neoformans*; cryptococcosis; host immune response; immunodeficiency; sterylglucosides; immunoadjuvant; γ*δ* T cells; toll-like receptors; IFNγ; IL-17A; adjuvants, glucuronoxylomannan

Supplementary Figure 1. Experimental design scheme for Figure 1. CBA/J mice were administered isotype, anti-CD8, or anti-CD4 antibodies (day -32), which continued for the entirety of the experiment. Mice then received either *C. neoformans* (*Cn*) Δ*sgl1* or PBS (day -30). After 30 days, mice were challenged with *C. neoformans* WT (day 0). On days -15, -1, 7, 15, and 24, mice were assessed for T cell-derived cytokines via intracellular cytokine staining (ICS).

Supplementary Figure 2. Representative gating strategy for T cell-derived cytokine production in mouse lungs. Lungs were excised from euthanized mice and processed to make single-cell suspensions as described in the Materials and Methods for intracellular cytokine staining analysis. $CD4^+$ and $CD8^+$ T cells were gated from live, $CD45^+$ leukocytes for the production of IFNγ, IL-17A, and IL-13.

Supplementary Figure 3. Vaccination with *C. neoformans* Δ*sgl1* prevents a harmful type 2 T cell polarization state in immunocompetent, CD4-deficient, and CD8-deficient mice. CBA/J mice (n=3 mice/group/timepoint) were administered isotype (**A-B**), anti-CD8 (**C**), or anti-CD4 (**D**) antibodies. Mice then received either *C. neoformans* Δ*sgl1* (white symbols) or PBS (black symbols). After 30 days, mice were challenged with *C. neoformans* WT (day 0). On days -15, -1, 7, 15, and 24, mice were assessed for IL-13 via intracellular cytokine staining. Graphed data represents the mean +/- SD and is representative of two independent experiments. Significance was determined by a two-way ANOVA using Šídák's multiple comparisons test for *P* value adjustment, and significance is denoted as *, $P < 0.05$; ****, $P < 0.001$.

Supplementary Figure 4. IL-17A neutralization or effector cell depletion results in loss of host protection in *C. neoformans* Δ*sgl1*-vaccinated mice. **A**. CBA/J mice were depleted of either IFNγ or IL-17A in the presence or absence of $CD4^+$ or $CD8^+$ T cells, administered *C. neoformans* Δ*sgl1*, and assessed for survival over 30 days. **B-C**. Organ fungal burden was assessed in the lung, brain, spleen, kidney, and liver from mice that were neutralized of IL-17A (**B**) or IL-17A under conditions of either anti-CD4 or anti-CD8 immunodeficiency (**C**), challenged with *C. neoformans* WT strain, and survived until the experimental endpoint. **D**. Vaccinated and unvaccinated mice were challenged with *C. neoformans* WT strain and assessed for survival in the absence of neutrophils ($Ly6G^+$ cells). Graphed data represent the survival percentage of challenged mice (**A, D**) or mean ± SD of 3 mice/group/timepoint (**B-C**). Significance was determined by a two-way ANOVA using Šídák's multiple comparisons test for *P* value adjustment, and significance is denoted as *, $P < 0.05$; ***, $P < 0.005$; ****, $P < 0.001$ (**B-C**). Survival significance was determined by the Mantel-Cox log-rank test, and denoted on each graph: **A**: $, $P < 0.001$for Δ*sgl1*/anti-IFNγ vs. Δ*sgl1*/anti-CD4 + anti-IFNγ; **C**: &, $P < 0.001$ for Δ*sgl1*/Isotype → WT vs. Δ*sgl1*/anti-Ly6G → WT.

Supplementary Figure 5. Representative gating strategy for γδ T cells. Lungs were excised, processed into single cell suspensions, and assessed via flow cytometry as described in the Materials and Methods section. From live, $CD45^+$ leukocytes, γδ T cells were quantified as $TCR\gamma\delta^+$ $CD3^+$ cells.

Supplementary Figure 6. TCR$\delta^{-/-}$ mice fail to control *C. neoformans* Δ*sgl1* resulting in extrapulmonary dissemination. Organ fungal burden was assessed in the brain (**A**), spleen (**B**), kidney (**C**), and liver (**D**) in C57BL/6 and TCR$\delta^{-/-}$ mice administered *C. neoformans* Δ*sgl1* on the timepoints indicated. Data shown represent the mean ± SD of 3 mice/group/timepoint. Significance was determined by a two-way ANOVA using Šídák's multiple comparisons test for *P* value adjustment, and significance is denoted as **, $P < 0.01$; ***, $P < 0.005$; ****, $P < 0.001$.

Supplementary Figure 7. γδ T cells robustly respond to HK *C. neoformans* Δ*sgl1* via the production of IFNγ and IL-17A. γδ T cells were purified from the spleens of uninfected C57BL/6 mice via MACS separation kit and cultured *ex vivo* with (+) or without (-) plate bound anti-TCRγ*δ*, stimulated with PBS, HK *C. neoformans* WT, HK *C. neoformans* Δ*sgl1*, or HK *C. neoformans* Δ*cap59*Δ*sgl1*, and assessed for IFNγ (**A**) and IL-17A (**B**) production on days 1, 3, and 5 post stimulation. Graphed data represents the mean +/- SD. Significance was determined by a two-way ANOVA using Šídák's multiple comparisons test for *P* value adjustment, and significance is denoted as *, $P < 0.05$; **, $P < 0.01$; ***, $P < 0.005$; ****, $P < 0.001$.

Supplementary Figure 8. Role of toll-like receptor 2 (TLR2) on HK *C. neoformans* Δ*sgl1*-induced cytokine production by γδ T cells *ex vivo*. γδ T cells were purified from the spleens of uninfected C57BL/6 ($TLR2^{+/+}$) or $TLR2^{-/-}$ mice via MACS separation kit and co-cultured *ex vivo* with plate-bound anti-TCRγ*δ*. $TLR2^{+/+}$ γδ T cells were cultured with $TLR2^{-/-}$ antigen presenting cells (APCs) or $TLR2^{-/-}$ γδ T cells were cultured with $TLR2^{+/+}$ APCs, stimulated with PBS, HK *C. neoformans* WT, HK *C. neoformans* Δ*sgl1*, or HK *C. neoformans* Δ*cap59*Δ*sgl1*, and assessed for IFNγ (**E**) and IL-17A (**F**) on days 1, 3 and 5 post stimulation. Graphed data represent the mean +/- SD. Significance was determined by a two-way ANOVA using Šídák's multiple comparisons test for P value adjustment, and denoted as *, $P < 0.05$; ****, $P < 0.001$.

Supplementary Figure 9. Toll-like receptor 2 (TLR2) is required for pulmonary clearance and containment of *C. neoformans* Δ*sgl1*. A. C57BL/6 and TLR2$^{-/-}$ mice (n=10 mice/group) were inoculated with *C. neoformans* Δ*sgl1* and assessed for survival. **B**. Organ fungal burden was assessed in the lung, brain, spleen, kidney, and liver of C57BL/6 and TLR2$^{-/-}$ mice (n=3 mice/group) at the experimental endpoint after inoculation with *C. neoformans* Δ*sgl1*. Graphed data represent mice survival percentage (**A**) or the mean +/- SD (**B**). Significance was determined by a two-way ANOVA using Šídák's multiple comparisons test for *P* value adjustment (**B**). Significance is denoted as **, $P < 0.01$; ***, $P < 0.005$.

Supplementary Figure 1

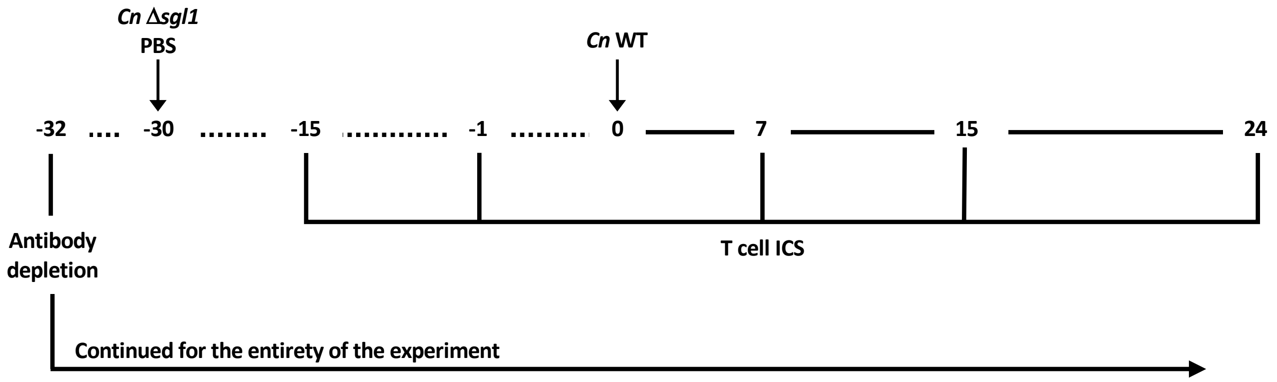

Supplementary Figure 2

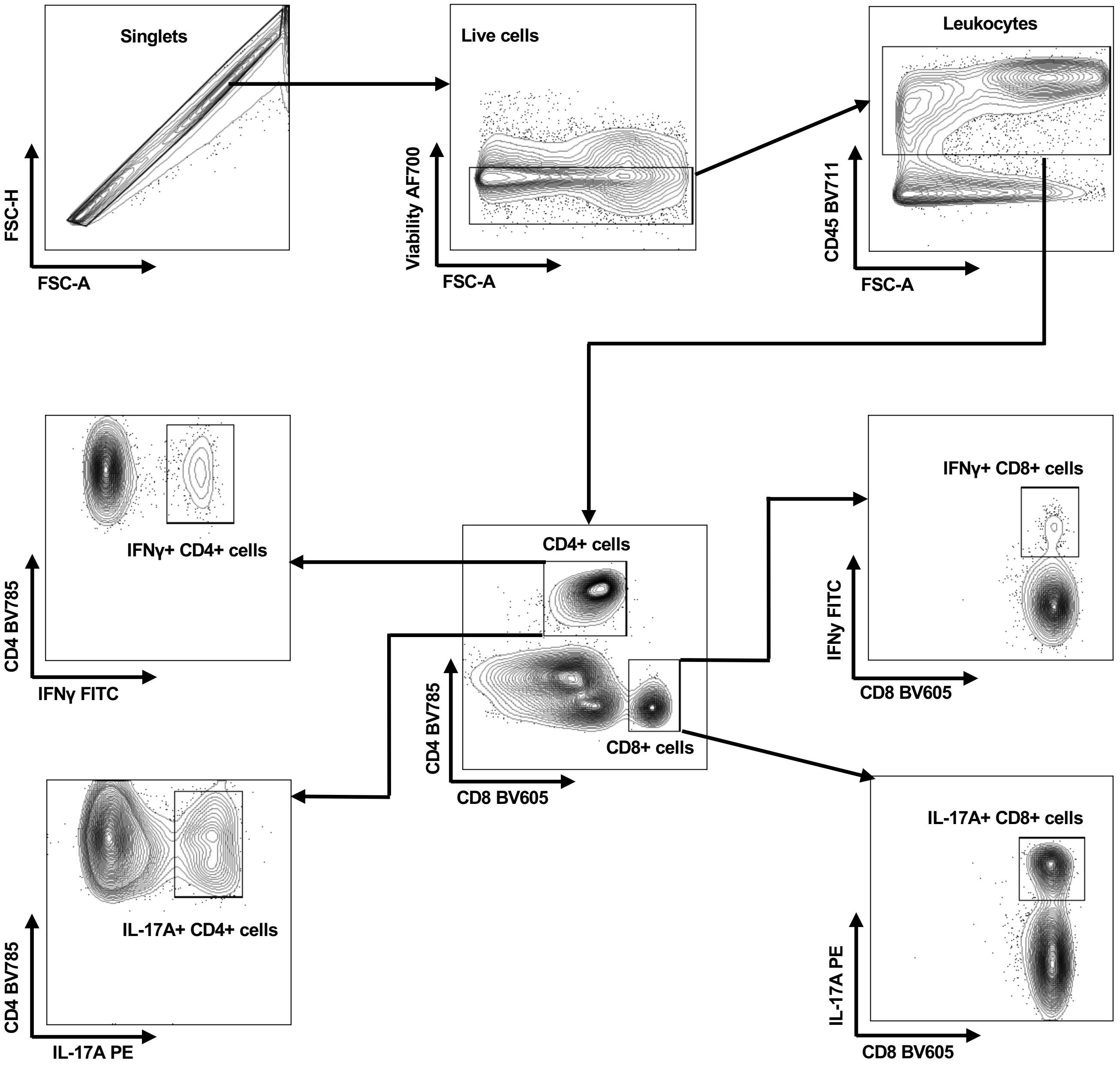

Supplementary Figure 3

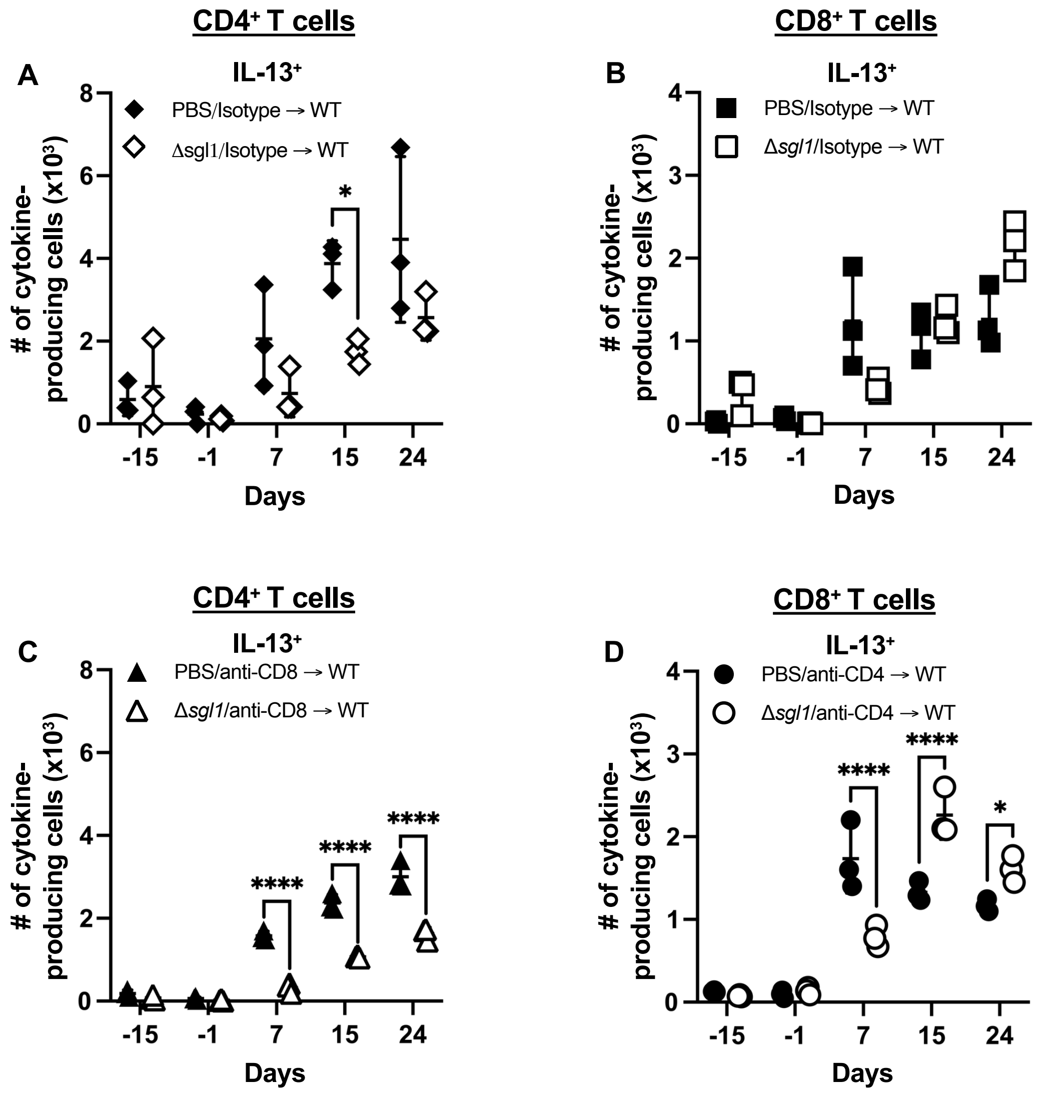

Supplementary Figure 4

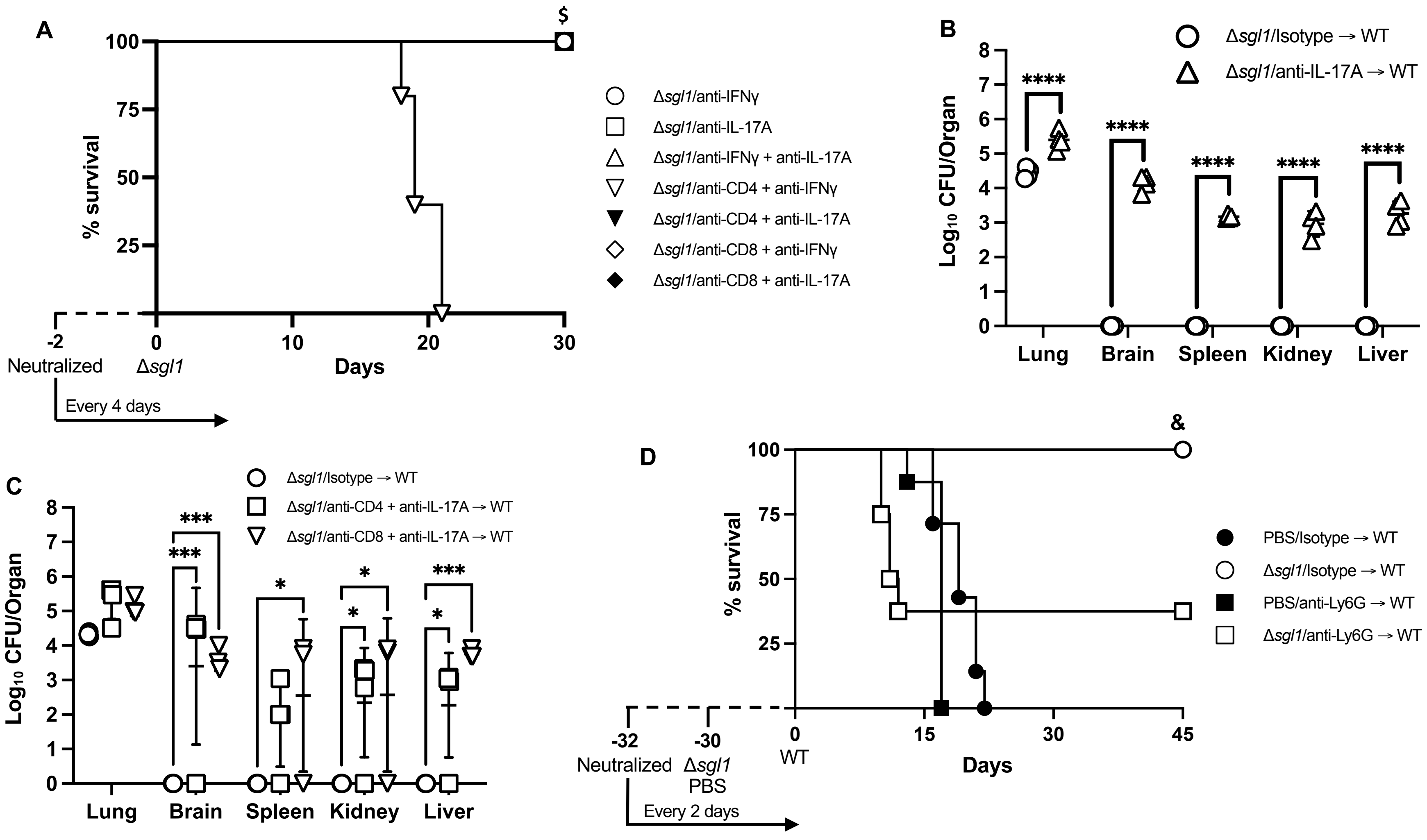

Supplementary Figure 5

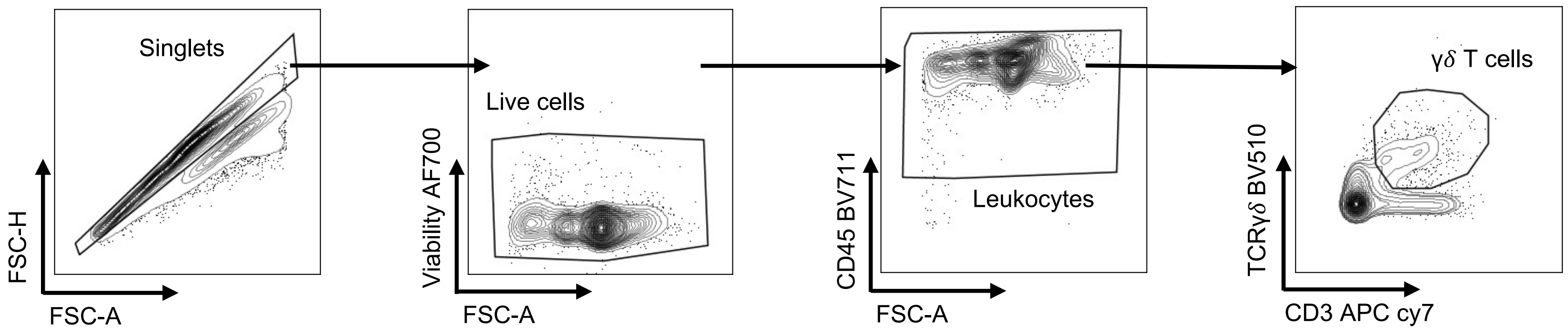

Supplementary Figure 6

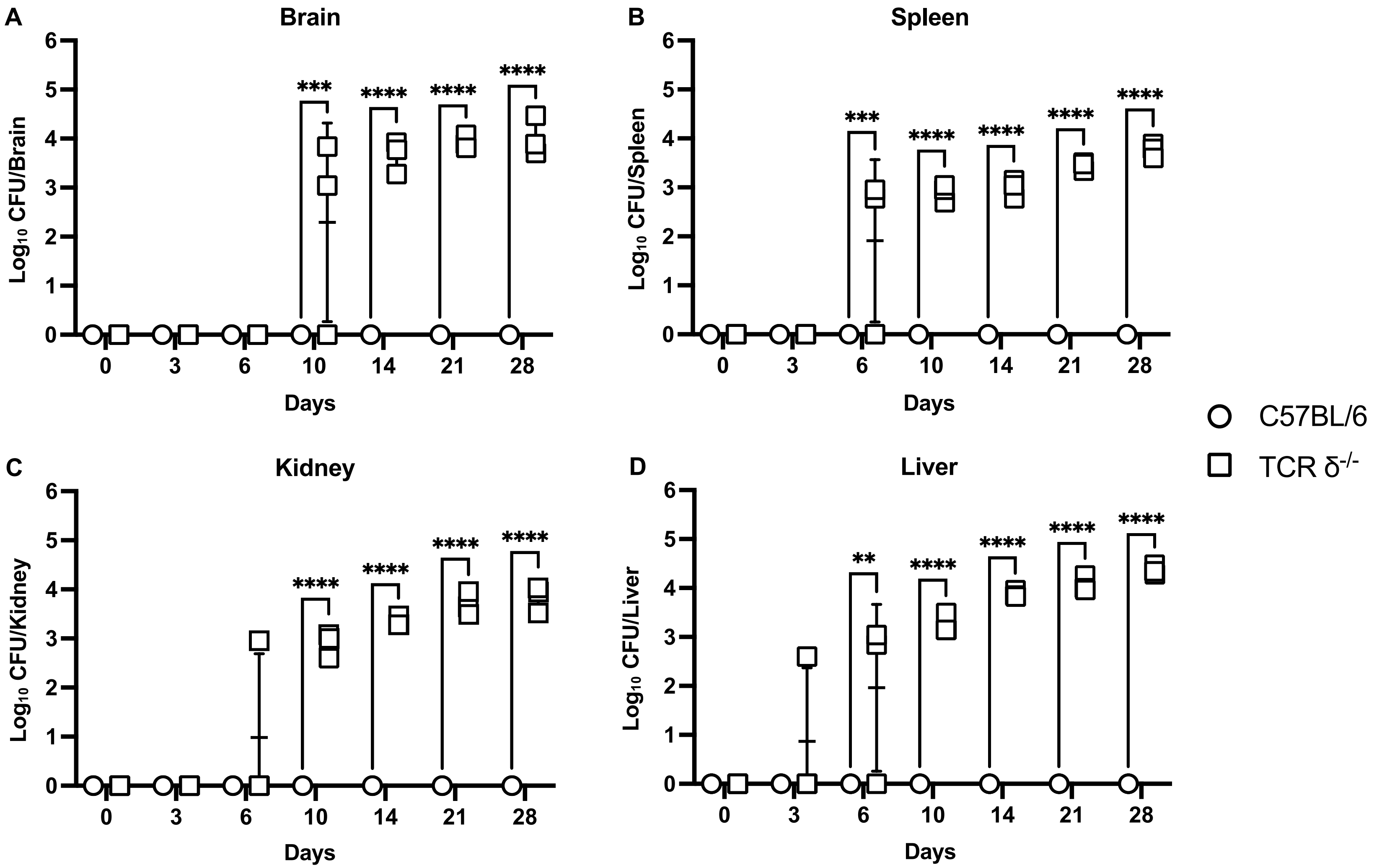

Supplementary Figure 7

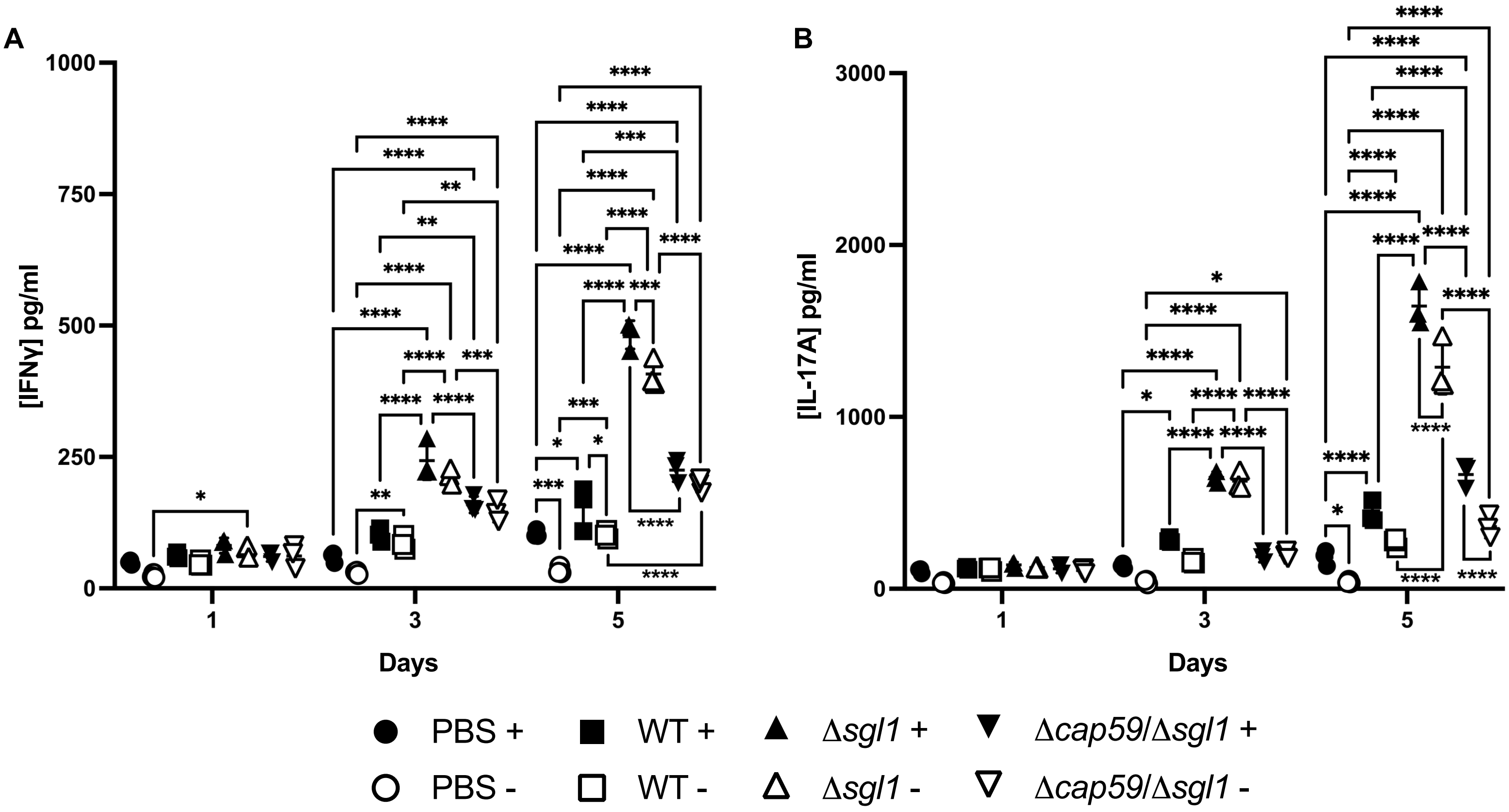

Supplementary Figure 8

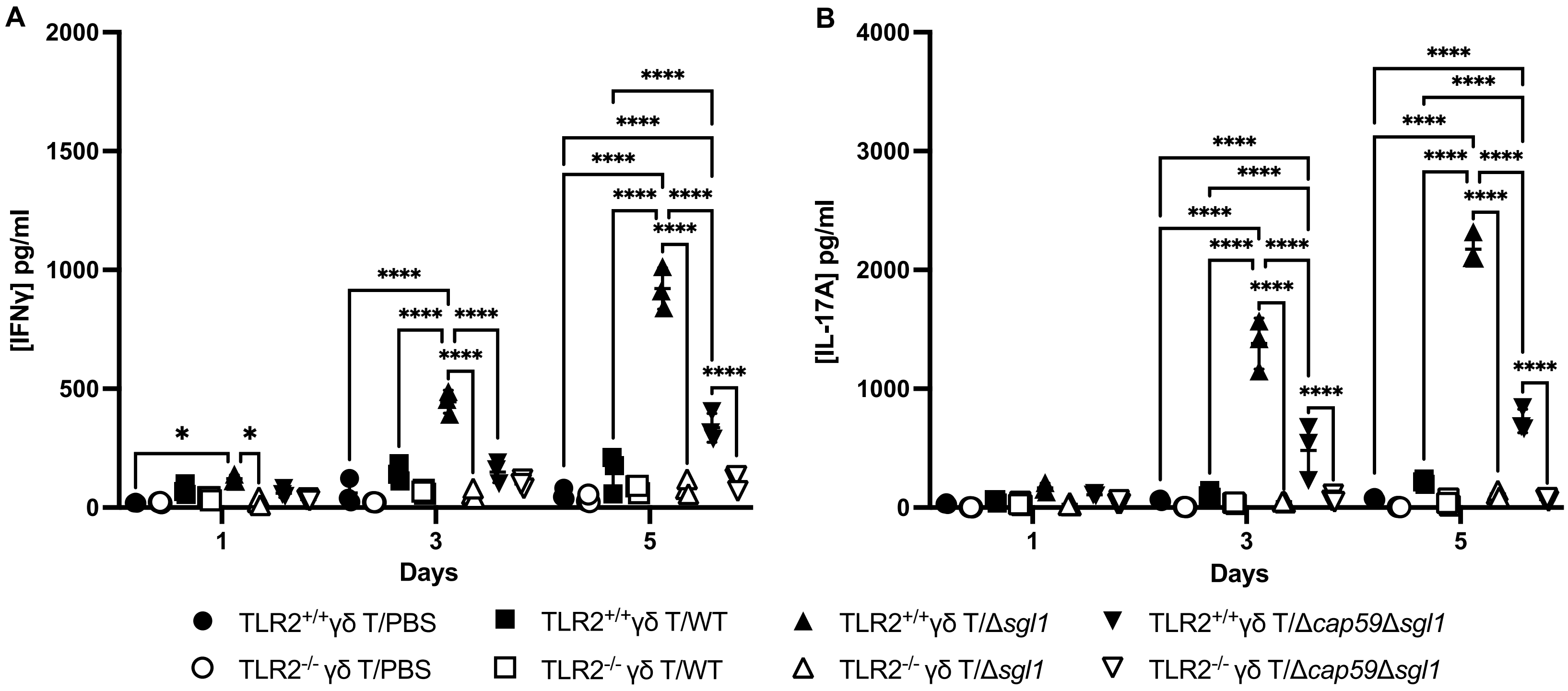

Supplementary Figure 9

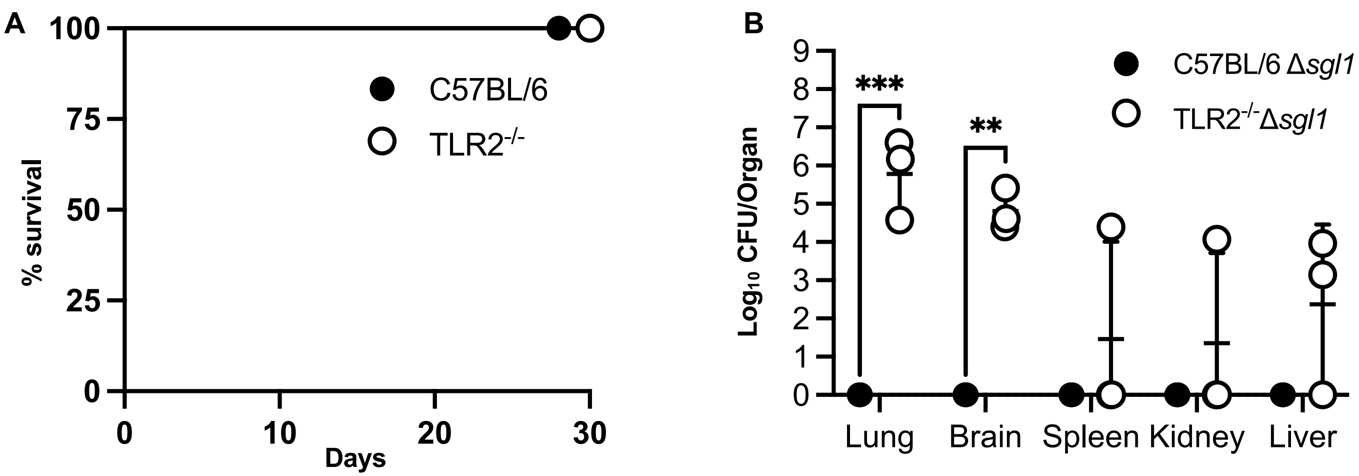

Supplement: Supplementary file 1 — Supplementary information [file 41385_2022_570_MOESM1_ESM.pdf]
